# Imaging Protein Aggregates in Parkinson’s Disease Serum Using Aptamer-Assisted Single-Molecule Pull-Down

**DOI:** 10.1021/acs.analchem.3c02515

**Published:** 2023-10-02

**Authors:** Yu P. Zhang, Evgeniia Lobanova, Derya Emin, Sergey V. Lobanov, Antonina Kouli, Caroline H. Williams-Gray, David Klenerman

**Affiliations:** † Department of Chemistry, University of Cambridge, Lensfield Road, Cambridge CB2 1EW, United Kingdom; ‡ UK Dementia Research Institute at Cambridge, Cambridge CB2 0XY, United Kingdom; § Medical Research Council Centre for Neuropsychiatric Genetics and Genomics, Cardiff University, Cardiff CF24 4HQ, United Kingdom; ∥ Department of Clinical Neurosciences, University of Cambridge, Cambridge CB2 0PY, United Kingdom

## Abstract

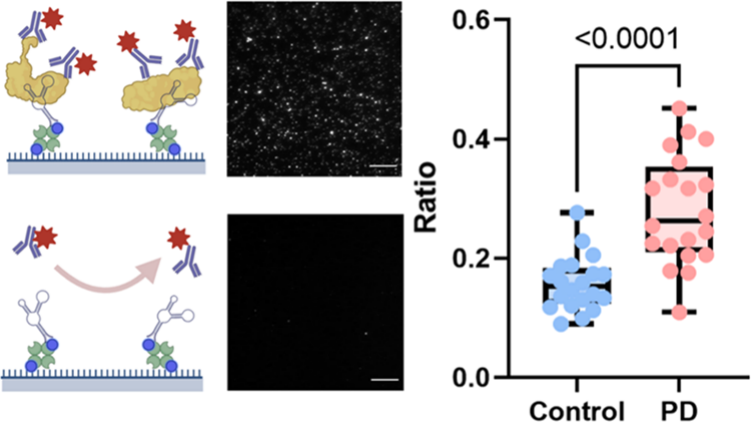

The formation of soluble α-synuclein (α-syn) and amyloid-β (Aβ) aggregates is associated with the development of Parkinson’s disease (PD). Current methods mainly focus on the measurement of the aggregate concentration and are unable to determine their heterogeneous size and shape, which potentially also change during the development of PD due to increased protein aggregation. In this work, we introduce aptamer-assisted single-molecule pull-down (APSiMPull) combined with super-resolution fluorescence imaging of α-syn and Aβ aggregates in human serum from early PD patients and age-matched controls. Our diffraction-limited imaging results indicate that the proportion of α-syn aggregates (α-syn/(α-syn+Aβ)) can be used to distinguish PD and control groups with an area under the curve (AUC) of 0.85. Further, super resolution fluorescence imaging reveals that PD serums have a higher portion of larger and rounder α-syn aggregates than controls. Little difference was observed for Aβ aggregates. Combining these two metrics, we constructed a new biomarker and achieved an AUC of 0.90. The combination of the aggregate number and morphology provides a new approach to early PD diagnosis.

## Introduction

There is currently no laboratory-based diagnostic test using biofluid samples for Parkinson’s disease (PD) in medical practice. Diagnosis of PD currently relies on the detection of the emergence of motor symptoms.^1,2^ By the time of diagnosis, around 60% of dopaminergic neurons (SNpc) are lost in the substantia nigra.^3^ Affected individuals experience inexorable physical and cognitive decline over several years and require increasing levels of support and care.^3,4^ While some of the motor symptoms of PD are responsive to treatment with dopamine-based therapies, balance problems and cognitive decline represent major therapeutic challenges, with no available therapies to modify the course of neurodegeneration and slow their progression. Within the first 10 years from diagnosis, postural instability and falls affect two-thirds of patients, while dementia affects nearly half.^5^ These impairments devastatingly affect the quality of life of both the patients and their families. There is an unmet need to develop methods that allow earlier detection of disease before the manifestation of symptoms since any available disease-modifying treatment is likely to be more effective if applied earlier. Furthermore, there is a need for readily available blood-based biomarkers that track disease progression to act as surrogate markers which will facilitate improved clinical trials of novel therapies.

Soluble α-synuclein (α-syn) and β-amyloid (Aβ) aggregates in the blood are potential biomarkers for PD.^6−8^ The deposition of insoluble α-syn aggregates inside brain neurons, which are known as Lewy bodies, is a pathological hallmark of PD. It is also common for PD to have copathology with other protein aggregates: cortical Aβ plaques and tau neurofibrillary tangles also occur in PD, which may contribute to the rapid cognitive decline and dementia during the disease progression.^9^ Unlike these insoluble species, smaller soluble aggregates form earlier in the protein aggregation process. They may be secreted from neurons or released by dying cells and are subsequently cleared from the brain’s interstitial fluid into the blood via the glymphatic system.^10^ Both *in vitro* and animal model studies have suggested their potential neurotoxicity.^11−14^ Therefore, soluble aggregates in the blood are potentially biomarkers capable of identifying PD pathology at an early stage. Previously, techniques including enzyme-linked immunosorbent assay (ELISA), Western blots (WB), immunomagnetic reduction (IMR), and Luminex have been utilized to examine the protein aggregates in PD-derived biofluids.^15−17^ However, protein aggregates are heterogeneous in both size and structure. Since this heterogeneity is linked to the toxicity of aggregates,^11,14,18^ none of the bulk measurements performed using the abovementioned methods are optimal: they cannot measure the size and shape of individual aggregates. Therefore, methods capable of detecting single aggregates provide important diagnostic information.

Single-molecule super-resolution imaging offers an approach to fingerprinting single populations without performing parallel bulk measurements. It overcomes the resolution barrier from the diffraction limit (∼200 nm) and reveals detailed morphological features of these heterogeneous aggregates, providing additional metrics that might identify marginal differences between aggregates from age-matched controls and early stage patients, due to increased aggregation in disease. We recently investigated the size difference between soluble β-sheet-rich aggregates found in the cerebrospinal fluid (CSF) and serum samples from PD patients and controls:^8^ larger aggregates (length >150 nm) were found to be more abundant in PD serum samples in comparison to controls. Furthermore, the proportion of α-syn aggregates (α-syn/(α-syn + Aβ)) was significantly higher in PD, discriminating PD and control cases with an accuracy of 98.2% (AUC = 0.982).^8^ However, there are still a few limitations: (1) The sample preparation was time-consuming and requires a large volume of around 1 mL per patient. (2) Quantification of the super-resolution images was still quite basic since only the size but not the shape of aggregates was assessed, resulting in insufficient discrimination for individual diagnosis. (3) DNA point accumulation in nanoscale topology (DNA-PAINT)-based super-resolution imaging, used previously, is slow and cannot eliminate false-positive signals due to nonspecific imager–sample interaction.

Here, we introduce the APSiMPull assay to characterize α-syn and Aβ aggregates in serum samples from PD and control groups. Single-molecule pull-down (SiMPull) is a versatile platform for highly sensitive and specific single-molecule imaging.^19−21^ Using this method, Je et al. revealed more α-syn aggregates in PD post-mortem brain samples compared to controls.^19^ In this work, we modified the assay by introducing the β-sheet-specific T-SO508 aptamer^22^ and direct Stochastic Optical Reconstruction Microscopy (dSTORM) super resolution fluorescence imaging.^23^ The application of T-SO508 provides the imaging surface with affinity to both soluble α-syn and Aβ aggregates due to their common β-sheet-rich structure. Subsequent dSTORM imaging provides morphological information on the captured aggregates, offering additional metrics for aggregate characterization. Results from this study extend our previous work and validate the characterization of α-syn and Aβ aggregates in serum as a potential PD biomarker.^8^ Critically, a clear morphological difference between α-syn aggregates in PD and control serum was also observed. By combining the information from both diffraction-limited and super-resolution imaging, we found that a combined biomarker showed very promising performance in terms of discriminating PD cases from controls. This study addresses the limitations of our previous work and also offers a generic analysis pipeline for biomarker discovery in other neurodegenerative diseases by, for the first time to our knowledge, combining measurement of the aggregate number with super-resolution imaging of the aggregates’ shape and size.

## Experimental Section

### Participants

Patients with idiopathic Parkinson’s disease (diagnosed according to UK PD Brain Bank Criteria, and within a year of diagnosis) were enrolled at the Cambridge Parkinson’s Disease Research Clinic at the John Van Geest Centre for Brain Repair, University of Cambridge, U.K. Age- and sex-matched participants without neurological disease were recruited from the NIHR Cambridge Bioresource (http://www.cambridgebioresource.org.uk). Demographic data were collected from all participants. Participants with a diagnosis of PD were assessed using the Movement Disorder Society Unified Parkinson’s Disease Rating Scale (MDS-UPDRS) and completed neuropsychological testing, including the Addenbrooke’s Cognitive Examination (ACE-III). The PD stage was determined using the Hoehn and Yahr scale (Table 1). The primary cohort comprised 20 PD cases and 20 controls (Table 1). Samples from a second cohort of 9 patients and 9 controls (Table 2) were used to validate the method via an inverted imaging strategy (antibody capture but aptamer detection). Ethical approval was obtained from the East of England–Essex Research Ethics Committee (16/EE/0445) and written informed consent was provided by all participants.

**Table 1:** Demographic and Clinical Characteristics of the Primary Cohort Investigated in the APSiMPull Experiment

	control	PD	*p* value
sample size	20	20	1
age (years)	60.9 ± 13.9	65.8 ± 7.1	0.2
sex (% male)	35%	50%	0.5
ACE-III		93.0 ± 5.4	
disease duration (years)		0.5 ± 0.3	
Hoehn & Yahr		1.7 ± 0.6	
MDS-UPDRS III		26.6 ± 11.6	
MDS-UPDRS Total		48.7 ± 20.8	
storage duration (years)	1.2 ± 0.5		

**Table 2:** Demographic and Clinical Characteristics of the Secondary Cohort Investigated in the Aptamer SiMPull Experiment^a^

	control	PD	*p* value
sample size	9	9	1
age (years)	66.5 ± 9.8	65.1 ± 6.0	0.7
sex (% male)	56%	67%	1
ACE-III		95.4 ± 3.0	
disease duration (years)		0.6 ± 0.5	
Hoehn & Yahr		1.8 ± 0.7	
MDS-UPDRS III		24.8 ± 9.2	
MDS-UPDRS total		44.3 ± 19.7	
storage duration (years)	0.2 ± 0.1		

a Values represent the mean ± SD. Variables were compared using the permutation (exact) test except for the sample size for which the binomial test was used (*p < 0.05).

### APSiMPull Experiment Protocol

The protocol is adapted from our previous reports with slight modifications.^11^ Once the SiMPull coverslips were taken from the desiccator and equilibrated to room temperature, 10 μL of 0.2 mg/mL NeutrAvidin (Thermo Scientific, 31000) diluted in PBS-T (0.05% Tween 20, diluted from potassium-rich Rockland MB-075-1000) was added to each well and incubated for 5 min. The wells were then washed twice with 10 μL of PBS-T by pipetting the liquid in and out. 10 μL of 10 nM biotinylated T-SO508 aptamer was added to each well and incubated for 10 min. Aptamers were annealed following the published method.^22^ For control/validation experiments, the biotinylated aptamer was replaced with either control aptamer (10 nM), antibodies (10 nM) or BSA (0.1 mg/mL in PBS-T). Each well was then washed twice with 10 μL of PBS-T. 10 μL of samples were then added to the wells and incubated for 90 min (serum) or 30 min (synthetic aggregates). For detection of both α-syn and Aβ, 10 μL of 5 nM detection antibodies were added to the well and incubated for 10 min before washing 3 times with PBS-T. For the validation experiment, 10 nM of AF-647 T-SO508 supplemented with 1 nM MgCl_2_ was added and incubated for 10 min before washing 3 times with PBS-T. For dSTORM control experiment, a higher concentration (10 nM) and longer incubation (15 min) of IgG isotype controls were used to create nonspecific signals for assessing the morphological information of antibodies. Unless mentioned specifically, all of the dilutions of antibodies/aptamers were made in PBS-T. For diffraction-limited imaging, 5 μL of PBS was added to each well and retained during image acquisition. For dSTORM imaging, another 3 layers of PDMS chambers were stacked onto the coverslip to increase the well capacity. 16.5 μL of dSTORM buffer (50 mM PBS-Tris, 0.5 mM glucose, 1.3 μM glucose oxidase, 1.1 μM catalase, and 25 mM mercaptoethylamine (MEA), pH 8.0) was then added to each well. MEA was added to the buffer immediately before imaging. To maintain the pH during imaging, the top of the chambered coverslip is sealed using a second cleaned coverslip. The edges of the integrated coverslip complex were further treated with nail polish and parafilm to reduce oxygen penetration. All patients’ samples were characterized in duplicate. See the Supporting Information (SI) for the details of the sample, coverslips, and probe preparations.

### Imaging Setup

Imaging was performed by using a home-built total internal reflection fluorescence (TIRF) microscope. A Nikon Ti2 Eclipse inverted microscope is integrated with a 100× 1.49 NA oil-immersion objective (UPLSAPO, 100×, TIRF, Olympus) and a perfect focus system. An excitation laser beam (Oxxius, 638 nm) was circularly polarized by a quarter-wave plate (WPQ05M-405, Thorlabs) and focused onto the back focal plane of the objective. The fluorescence emission was collected using the same objective and separated by a dichroic beamsplitter (Di01-R405/488/561/635, Semrock), with filtering performed by a long-pass emitter (BLP01-635R-25, Laser 2000). Emission is imaged onto an air-cooled EMCCD camera (Photometrics Evolve, EVO-512-M-FW-16-AC-110) with frame transfer mode (electron-multiplying Gain of 11.5e-1/ADU and 250ADU/photon). The open-source software Micro-Manager 1.4 was employed to automate image acquisition. 638 nm laser (iBeam-Smart, Toptica) was used to excite Alexa 647 dyes. For diffraction-limited imaging, 1.5 mW of laser power was applied, and images were acquired with an exposure time of 50 ms and frame number of 50. For dSTORM imaging, 150 mW of laser power was applied, and images were acquired with an exposure time of 15 ms and frame number of 6000–8000. The morphological information on aggregates starts to have a stable distribution for a frame number higher than 5000 (see Figure S11 in the SI). The camera was operated with pre-exposure nonoverlapping mode to precisely control the exposure time. Continuous illumination by 405 nm laser (LBX-405–50-CIR-PP, Oxxius) at 10 mW was applied. The pixel size of the image was measured as 103.5 nm. Each field of view (FoV) contains an area of around 2500 μm^2^. For each patient, a total number of at least 28 images (diffraction-limited) and 6 images (dSTORM) were taken from 2 replicates.

### Data Analysis

The diffraction-limited data were analyzed using in-house software called Path-Connected Aggregate Recognition (PCAR). Details of the software can be found in the Supporting Information. dSTORM data was analyzed using established ImageJ plug-ins. The drift correction, image reconstruction, and morphology analysis were performed by mean shift algorithm,^24^ ThunderSTORM,^25^ and morphology library,^26^ respectively. A custom-written Matlab code was used to integrate mentioned plug-ins and automate data analyzing. A detailed explanation is included in the Supporting Information. All data were first assessed using a Kolmogorov–Smirnov test to ensure normality with α = 0.05 (see Supporting Information Table S1 for details). For normally distributed data, a two-tailed *t* test with Welch’s correction was employed. Otherwise, Wilcoxon rank-sum (Mann–Whitney U test) was used. Statistical significance was indicated when *p* < 0.05. Receiver operating characteristic (ROC) analysis was performed using the Wilson method, which is commonly used to examine diagnosis-related data.^27,28^ The code for rendering the data is publicly available at https://github.com/LobanovaEG-LobanovSV/PCAR (diffraction-limited) and Issues YPZ858/Super-res-code (github.com) (super-resolution)

## Result and Discussion

### Establishment of the APSiMPull Assay for Serum Aggregate Detection

**Figure 1:**
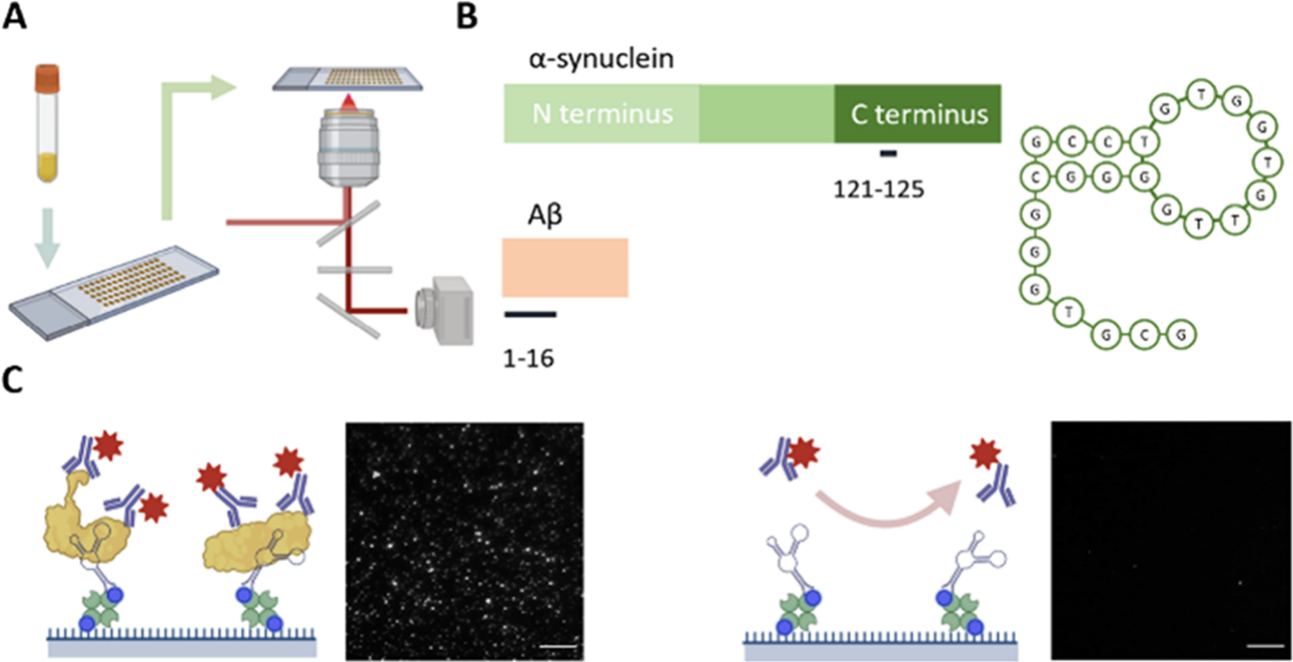
Workflow of APSiMPull. (A) Serum collected from patients was directly loaded on the assay and processed using a single-molecule fluorescence microscope. (B) Aptamer and antibodies used in this work. T-SO508 aptamer recognizes the β-sheet structure of both soluble α-syn and Aβ aggregates; 211 antibodies recognize the epitope aa. 121–125 of α-syn and 6E10 recognizes aa. 1–16 of Aβ. (C) Illustration of working principles of APSiMPull. The biotinylated aptamer is immobilized on the surface via NeutrAvidin–biotin interaction and used for target capturing. Once the target is captured, detection antibodies are added for immunostaining. If no sample is presented, the passivated surface will reject the nonspecific binding from detection antibodies to make the measurement specific. Scale bar: 10 μm.

**Figure 2:**
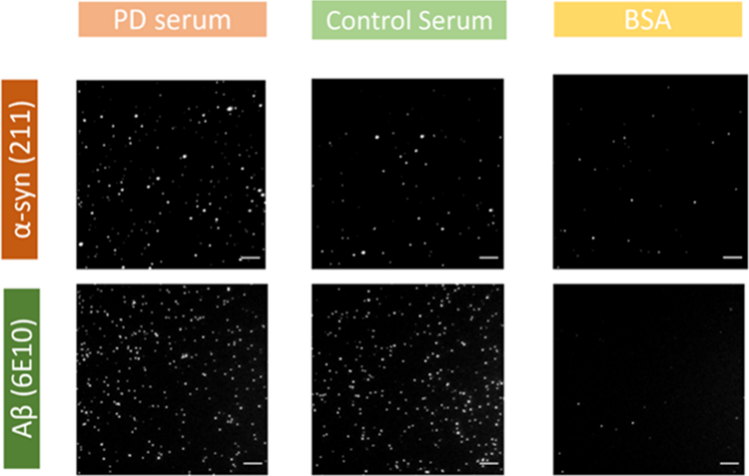
Representative detection of α-syn and Aβ aggregates in human serum. 211 and 6E10 antibodies are used to detect α-syn and Aβ aggregates. The representative data shown were calculated by averaging images taken from the respective samples. The same contrast was applied to each row or channel of images. BSA was used as the negative control. Scale bar: 5 μm.

Briefly, aptamers immobilized on glass coverslips were used to selectively capture the aggregates from the samples, as shown in Figure 1. Once the target was captured on the surface, fluorescently labeled antibodies were added for single-molecule detection. Polyethylene glycol (PEG) passivation prevents the fluorescence signal raised from nonspecific antibody bindings. Several modifications were made to the APSiMPull assay compared with the first reported antibody SiMPull: (1) Instead of using antibodies, a β-sheet-specific T-SO508 aptamer was used to capture targets. The aptamer was proven to be an efficient analogue to an antibody in surface-based biomolecule/cell capture assays.^29,30^ The T-SO508 aptamer has been widely used to investigate β-sheet-rich species in human biofluids and used as either a capture or a detection aptamer in biosensors/assays.^8,11,31−34^ This aptamer has a conformation-specific affinity to the α-syn and Aβ aggregates and provides a complementary binding mechanism to epitope-specific antibodies.^22^ The use of the aptamer not only offers specificity in the detection of aggregates but also avoids cross-talk with autoantibodies present in human biofluids. (2) No secondary antibodies are utilized in this work. The directly labeled primary antibodies simplify the experimental procedure and avoid nonspecific signals from additional antibodies. Moreover, since the fluorescence signal emission is directly from the primary antibodies, the linkage error due to secondary antibodies is eliminated and makes super-resolution imaging more accurate. (3) A double-coating strategy was used to enhance surface passivation. This strategy requires two rounds of covalent coating using PEG with different molecular weights and is reported to achieve a superior passivation quality.^35^ Most reported SiMPull assays only use one round of PEGylation;^19−21^ however, this modification helps improve the surface quality further. Figure 2 demonstrates the representative images collected with APSiMPull.

The assay was initially validated using a dilution series of sonicated *in vitro* aggregates as well as a control aptamer (a DNA G-quadruplex without known specific affinity to aggregates).^36^ A clear concentration dependence for the number of detected spots was observed for both α-syn and Aβ aggregates until saturation, while the negative BSA controls only generated neglectable signals (see Supporting Information Figure S4). The capture control further validates specific capture by the aptamer: The aptamer control and no capture control showed much less signal than the correct T-SO508 capture aptamer (Supporting Information Figure S4). Unlike epitope-specific antibodies, which tend to bind to the aggregates regardless of the size and shape, the aptamer-coated surface showed a much higher affinity to smaller aggregates than mature/elongated fibrils (see Supporting Information Figure S5), aligning with previous reports and makes it more suitable for detecting soluble aggregates formed during early phases of the disease.^22^

### Aggregate Ratio as a Biomarker

**Figure 3:**
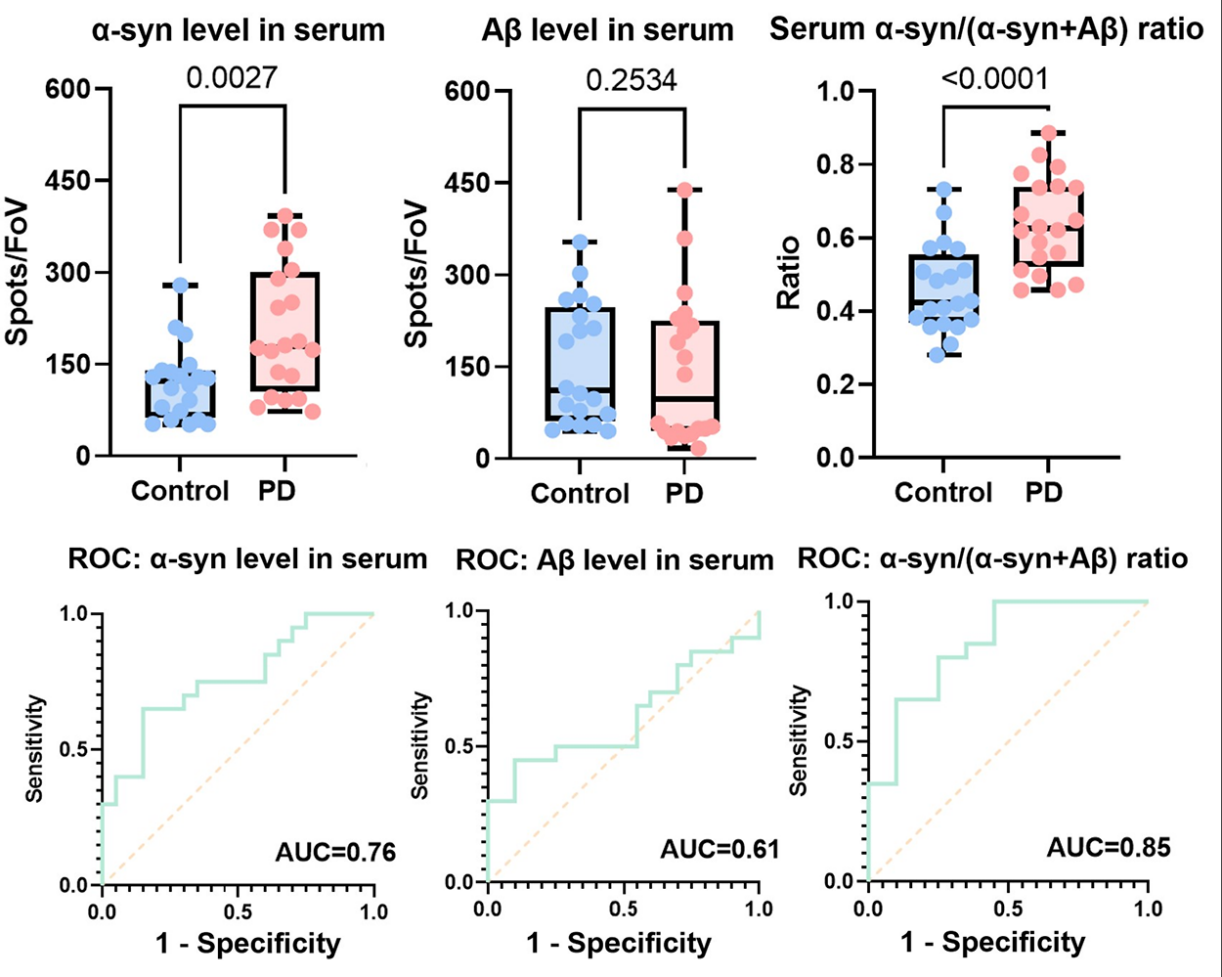
Single-molecule counting analysis of α-syn and Aβ aggregates in PD (*n* = 20) and control (*n* = 20) serum samples. Upper panel: The detection level of soluble α-syn and Aβ aggregates as well as their ratios in PD and control serum group are shown. Lower panel: ROC analysis was performed using the corresponding metrics. The level of soluble α-syn aggregates in serum achieved an AUC of 0.76, while Aβ achieved 0.61. The proportion of α-syn aggregates (α-syn/(α-syn + Aβ)) achieved an improved AUC of 0.85.

Following this successful initial validation, we then performed the characterization of the aggregates present in the serum. The detected levels of α-syn and Aβ aggregates from PD and control groups showed a clear difference. As shown in Figure 3, the abundance of soluble α-syn aggregates in serum samples from PD cases was generally higher than in serum from controls, while the level of soluble Aβ aggregates tended to be lower in PD samples. Briefly, for α-syn detection, the average detected spot per field of view (FoV) for PD and control serum was 200 ± 100 and 120 ± 60, respectively. For Aβ detection, the average detected spot per FoV for PD and control serum was 140 ± 120 and 150 ± 100, respectively. We also validated the capture specificity of the assay with human serum. Only the surface coated with the correct capture aptamer generates a strong signal (see Supporting Information Figure S6). The difference between the levels of detected soluble α-syn aggregates from the two groups was statistically significant (*p* = 0.003), while the difference between Aβ levels was not (*p* = 0.253). This finding is in agreement with our previous report using immunodepletion-assisted aptamer DNA-PAINT.^8^

These results suggest that more α-syn aggregates form during PD pathogenesis than Aβ aggregates. When only using the number of detected aggregates to discriminate the two groups, the α-syn level alone achieved an AUC of 0.76, while Aβ achieved an AUC of 0.61, which correlates with the p-values obtained. Better discrimination was achieved when the proportion of α-syn aggregates (α-syn/(α-syn + Aβ)) was used achieving an AUC of 0.85, in agreement with our previous report.^8^ Although the concentration of soluble α-syn aggregates in human blood has been extensively measured,^17,37,38^ the absolute values vary with detection methods and individual samples. Taking the ratio between α-syn and total aggregates (α-syn + Aβ), a measure of the proportion of α-syn aggregates, reduces the variation between samples as shown here and in our previous work.^8^

Although screened as an α-syn and Aβ oligomer-specific aptamer, T-SO508 is reported to bind both fibrillar and nonfibrillar aggregates at a single-molecule level, sharing a similar binding trend with Thioflavin T.^22,32^ In our previous studies, we differentiated monomeric and early stage (2 h) α-syn aggregates with this probe.^32^ The size of these aggregates is comparable to that of the ones we detected in serum. This aptamer is also reported to have a strong affinity to small Aβ aggregates when used as a capture agent in biosensors.^34^ Therefore, the aptamer-positive aggregates are mainly associated with species from the early phase of aggregation. No significant difference was observed in the total number of aggregates (α-syn + Aβ) detected in PD and control serum (Supporting Information, Figure S7). This suggests that the T-SO508 aptamer itself is not sufficient to quantify aggregate levels in serum due to its lack of protein specificity. This might also explain the marginal difference in aggregate levels between CSF samples from AD patients and controls, where this aptamer was employed as the only probe in the assay.^31^

### Single-Molecule Size Imaging for Aggregates

**Figure 4:**
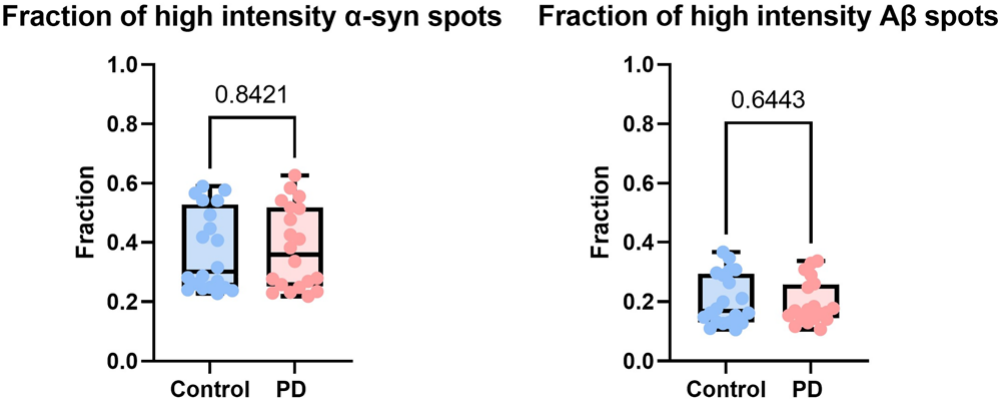
Single-molecule intensities of α-syn and Aβ aggregates in PD (*n* = 20) and control (*n* = 20) serum samples. High-intensity spots were defined as those with A.U. >25,000, as this value is close to the 75th percentile of the intensity profile of most samples. See the Supporting Information for details on intensity calculation.

Morphological information has been proven to be an additional metric useful for characterizing aggregates, especially when a small difference in number was observed.^8,31^ Unlike bulk measurements, single-molecule imaging allows the observation of individual protein aggregates, making the measurement of differences in aggregate size distribution possible. Previous reports have used single-molecule intensity as a correlation to the size of α-syn aggregates.^11,19^ Here, we measured the proportion of larger aggregates (A.U. >25,000, see the SI for intensity calculation) and found no difference between the two groups, as shown in Figure 4. However, this is an indirect measure of the size and shape of aggregates. Environmental quenching can also change the intensity profile of aggregates and reduce the correlation between the fluorescent intensity and size.

We went on to use dSTORM which is able to distinguish samples with a resolution of around 20 nm. Unlike previous reported DNA-PAINT-based assays,^8,31^ our dSTORM-based assay does not require freely diffusing imagers and hence avoids nonspecific signals contributed by both DNA–surface and DNA–sample interactions. The shorter camera exposure time of dSTORM (10–50 ms/frame),^23,39,40^ on the other hand, further speeds up the process when compared to DNA-PAINT-based methods. DNA-PAINT usually requires a longer camera exposure time (50–300 ms/frame)^8,11,31,32,41^ to reduce nonspecific signals caused by freely diffusing imaging strands. A commonly used glucose oxidase scavenger system was utilized in this work. To prevent the buffer pH from dropping, buffer-containing imaging chambers are tightly sealed to minimize oxygen penetration. We verified that careful sealing can keep the dSTORM functional for up to 12 h, which is sufficient for an assay to run and in agreement with a previously reported study (see Supporting Information Figure S9).^42^ We also verified that the morphology information on aggregates did not change during overnight imaging, while the intensity profile did (see Supporting Information Figure S9), indicating that dSTORM imaging is a stable approach to probe the morphological information of aggregates. dSTORM imaging was further validated by comparing images from serum samples, recombinant α-syn fibrils samples, and fluorescent IgG antibodies. Our results showed that the antibody spots are much smaller and rounder in comparison to all of the aggregate-containing serum samples, while fibrillar samples are much larger and elongated, as expected. Meanwhile, monomeric protein only has a single antibody binding epitope and is therefore identical to IgG-only signals.

### Morphological Information of Aggregates as a Supplementary Biomarker

Structural information on aggregates can serve as an additional metric for biomarker identification. Previous studies skeletonized aggregate images for analysis and mainly focused on a single parameter of length.^8,11,31,32^ In this study, we characterized the aggregate images directly without the need for skeletonization (see Supporting Information Figure S8). Two morphological parameters, aggregate perimeter (for size) and circularity (for shape) were used to quantify the morphological features of individual aggregates. The cumulative perimeter and circularity distributions and their relative differences were generated.

**Figure 5:**
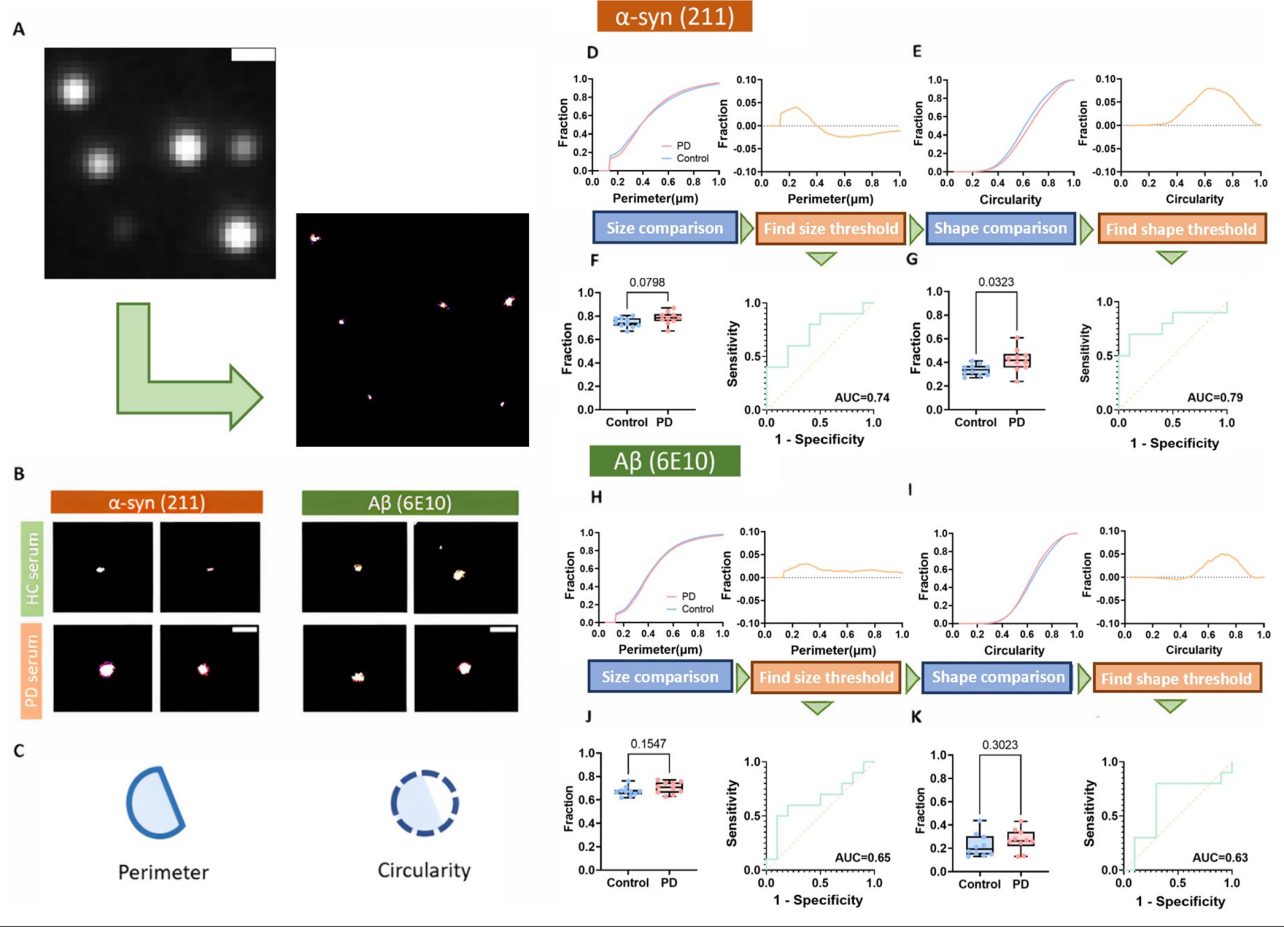
Morphology analysis of α-syn and Aβ aggregates in PD compared to HC serum using dSTORM. (A) Diffraction-limited image of the serum sample and corresponding dSTORM image. Using dSTORM, the finer morphologic information masked by the diffraction limit can be revealed. For this representative image, AF-647–211 antibody was used to visualize α-syn aggregates in serum. The scale bar is 0.75 μm. (B) Examples of super-resolved aggregates in serum samples. The scale bar is 0.5 μm. (C) Two parameters (perimeter and circularity) were used to quantify morphological information. (D–G) The cumulative perimeter/size distribution of α-syn for two groups (*n* = 20 for PD, *n* = 20 for controls). α-syn aggregates in PD serum are larger and rounder than those in control serum. The cumulative difference (control-PD) determines the optimal morphology thresholds. The discrimination performance is presented by ROC analysis and *t* tests, showing its potential as a discriminator. (H–K) An identical workflow from (D–G) was applied to Aβ (*n* = 10 for PD, *n* = 10 for controls). A smaller morphological difference between PD and the control was observed for Aβ. Further ROC and *t* tests suggest that these parameters are not sufficient to serve as a discriminator.

As shown in Figure 5, a clear morphological difference was observed between soluble α-syn aggregates from PD and the control serum. There was a smaller difference between these groups for soluble Aβ aggregates in serum. Besides greater abundance, PD patients have had a higher portion of larger and rounder α-syn aggregates in serum when compared with controls. To quantify this difference, we compared the averaged cumulative perimeter histograms of PD serum and controls and found that a maximum perimeter difference of 3.6% was observed at a perimeter of 0.24 μm. This critical perimeter was used as the first threshold to quantify the proportion of larger aggregates. We further compared the circularity of these larger aggregates.

**Figure 6:**
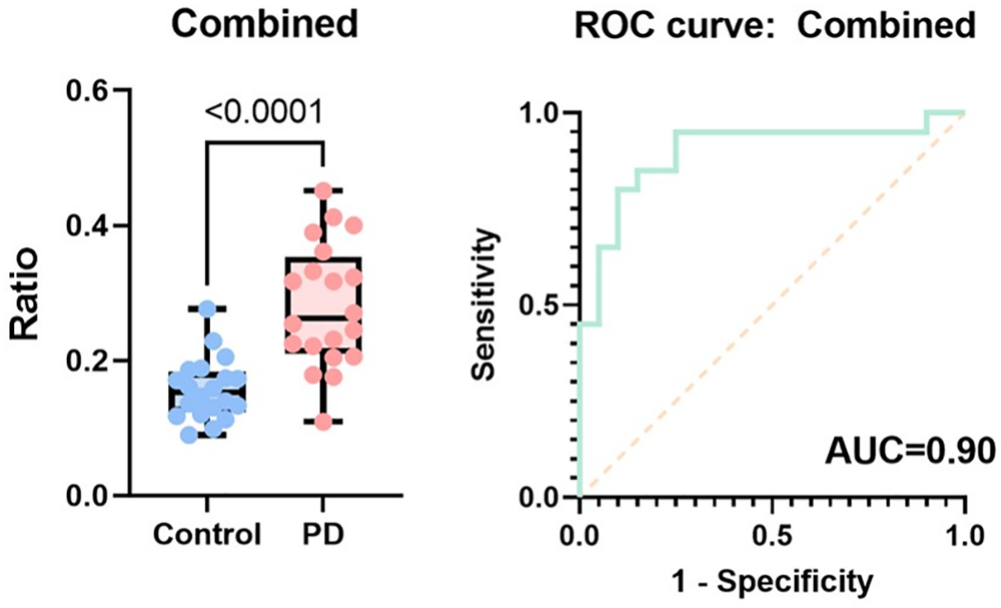
Combined discriminator and associated ROC analyses were performed for serum samples. The combined discriminator was constructed by multiplying the proportion of α-syn aggregates (α-syn/(α-syn+ Aβ)) and the fraction of morphologically distinct α-syn aggregates (*n* = 20 for PD, *n* = 20 for controls).

The relative differences between cumulative circularity histograms showed a maximum difference of 9.5% at a circularity of 0.64. We took these two parameters as our final thresholds to obtain the optimal morphological discrimination between PD and control serum (see Figure 5D–E). In contrast, little difference in these morphological features was observed for Aβ aggregates, as shown in Figure 5H–I. For Aβ, the relative differences between cumulative histograms showed that for perimeter and circularity, a maximum difference of 3.0% was observed at a perimeter of 0.31 μm and a difference of 5.0% at a circularity of 0.7. When setting these parameters as our threshold, we could not distinguish between PD and controls, as shown in Figure 5J–K. We finally constructed the best performance discriminator by multiplying the morphologically distinct α-syn aggregates with the proportion of α-syn aggregates (α-syn/(α-syn + Aβ)). This combined biomarker had an AUC of 0.90, as shown in Figure 6.

**Figure 7:**
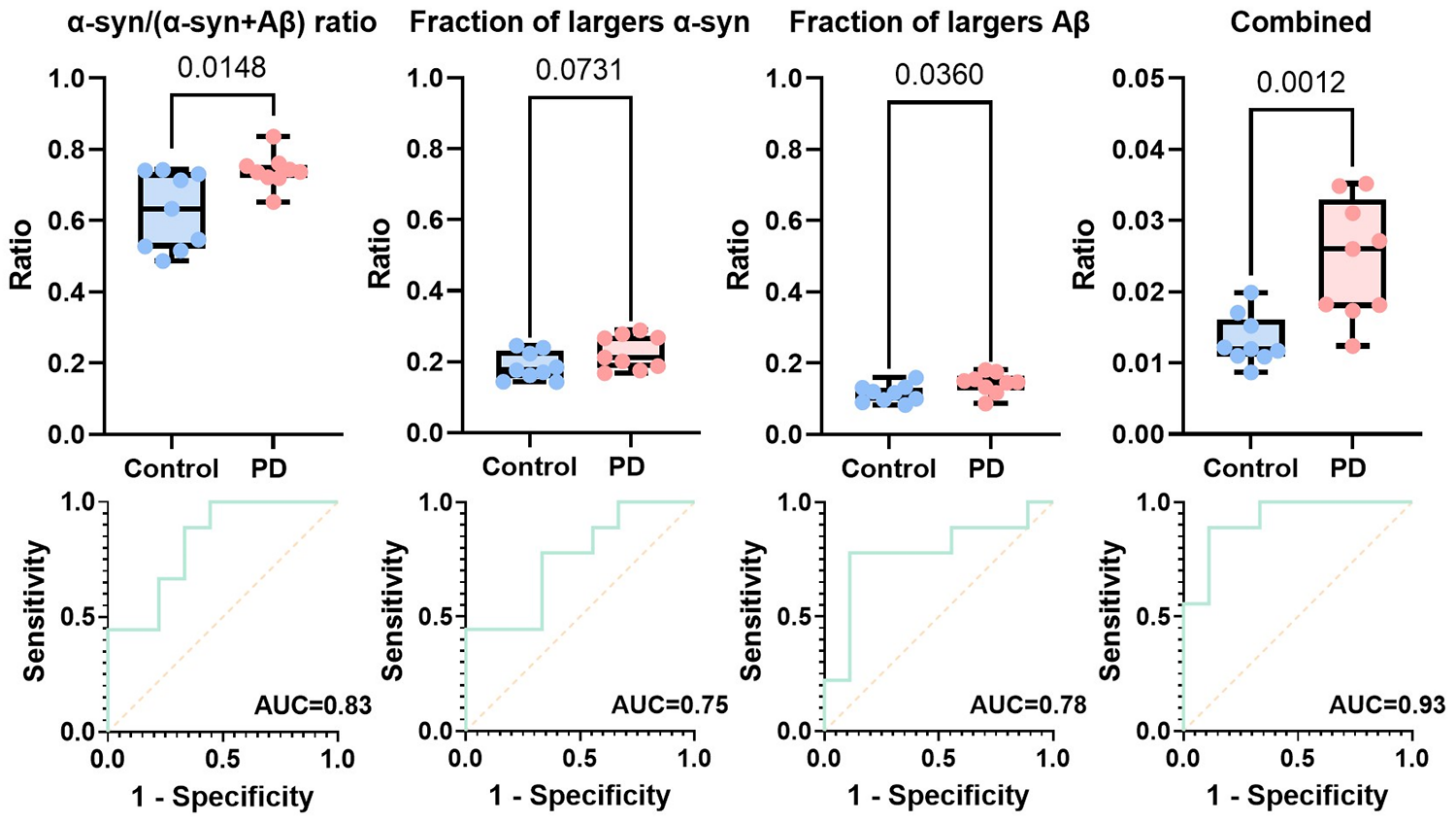
Discriminator and associated ROC analyses for the validation cohort. The combined discriminator was constructed by multiplying the proportion of α-syn aggregates (α-syn/(α-syn + Aβ)) and the percentage of brighter (larger) α-syn, as well as Aβ aggregates (*n* = 9 for PD and *n* = 9 for controls from the validation cohort). The intensity threshold used here is the same as that in Figure 4 (A.U. >25,000).

We examined a second set of PD and control samples to validate this method. An inverted detection strategy, where aggregates were captured using antibodies but imaged using aptamers, was used. As shown in Figure 7, the α-syn/(α-syn + Aβ) ratio is still able to distinguish two groups with an AUC of 0.83.

Interestingly, unlike antibody imaging, the intensities obtained via aptamer imaging showed a difference between PD and the control group. A larger fraction of high-intensity α-syn and Aβ aggregates was observed in PD samples, which matches the super-resolution imaging in the previous section. The smaller size (molecular weight of around 8 kDa while antibody is around 150 kDa) of these aptamers enables them to bind targets more effectively on small soluble aggregates, and hence, intensity is better correlated to the size of the aggregates. When combining the intensity and ratio information, we obtained an AUC of 0.93, which suggests that the combination of antibodies and the aptamer used in this study can provide good discrimination with flexibility in the way the assay is implemented.

## Conclusions

To summarize, we used APSiMPull to characterize soluble α-syn and Aβ aggregates in serum samples from PD cases and controls. Compared with controls, we found more T-SO508 aptamer-positive α-syn aggregates and slightly fewer Aβ aggregates in PD serum. The elevated concentration of soluble α-synuclein aggregates in PD serum signifies ongoing synucleinopathy within the patients. The reduced Aβ level detected in PD patients might also correspond to more pronounced neurodegeneration, as Aβ_1–40_ is the prevailing Aβ species known for its neuroprotective effects against metal-induced oxidative damage.^43,44^ Meanwhile, a higher proportion of larger and rounder α-synuclein (α-syn) aggregates is observed in PD serum compared to controls. This indicates the presence of detectable differences in the aggregate size in the serum of PD patients, likely due to the increased aggregation of α-syn in PD. However, minimal differences were observed for Aβ aggregates between the PD and control samples. Since the T-SO508 aptamer has an affinity to the β-sheet-rich aggregates with relatively small size,^22^ protein aggregates detected via this probe are associated with the early aggregation phase.^22,32^ Our previous work has shown that these smaller, soluble species are more toxic than aggregates from the later aggregation phase.^14,18^ Aggregates measured in blood may come from multiple sources. In PD, α-syn aggregates may originate in the periphery, from enteric neurons or red blood cells, or in the brain, and be exported into the blood via exocytosis-associated exosomes, and/or drained from the CSF and interstitial fluid via the glymphatic system.^37,45^ In our previous study, lower levels of CSF aggregates were associated with higher blood aggregates in controls, in contrast to a positive correlation in PD.^8^ This suggests that CSF is a route for clearing toxic aggregates from the brain which is more effective in people without PD. Characterizing protein aggregates in the brain and CSF with a similar method offers a chance to establish the link between aggregates presented in different environments.

Compared with our previously reported assay, APSiMPull provides an informative and faster method to examine the abundance and morphological features of protein aggregates. We validated that the proportion of α-syn aggregates (α-syn/(α-syn + Aβ)) can be used to discriminate between PD cases and controls. In comparison to our previous study, we obtained a comparable discrimination level (AUC = 0.85 vs AUC = 0.98) with a much shorter processing time and higher throughput (around 4 h per 20 samples vs 48 h per 20 samples). The faster acquisition of single-molecule localizations also allows more accurate morphological mapping of protein aggregates, as a slower imaging speed may result in an insufficient reconstruction of super-resolution images. The average localization bursts per molecule in this work is ∼20 times higher than our previous report,^8^ revealing better morphological details of protein aggregates (See Supporting Information Figure S12). In addition, we refined the quantification of super-resolution images of aggregates and found that the size (perimeter), as well as the shape (circularity) of α-syn aggregates, can serve as a supplementary discriminator. We achieved a superior AUC of 0.90 by combining metrics from both diffraction-limited and super-resolution images. Reported blood-based assays usually require additional postcollection processing of the sample (e.g., differential ultracentrifugation or ultrafiltration for exosome-based assays) to achieve similar results (from AUC = 0.77 to AUC = 0.98).^46,47^ Although seeding-based signal amplification assays have excellent specificity, they are largely applied to CSF and are rarely applied to blood samples.^48^ One immunomagnetic reduction (IMR) assay reported very promising results discriminating PD and control (AUC = 0.92 in serum and AUC = 0.99 in plasma) but was unable to generate the single-molecule profiles of α-syn aggregates.^37^ Other state-of-the-art methods, including Meso Scale Discovery (MSD) immunoassays and single-molecule array (Simoa) also share the same problem. Recent studies have revealed the potential significance of the aggregate structure in the disease status of PD.^8,11,49^ Profiling the single-molecule features of aggregates holds the potential to pinpoint key species, characterized by their morphology and composition. This, in turn, can contribute to the advancement of diagnostic and therapeutic strategies for PD. We further validated the method using an inverted imaging strategy, showing that the combination of the antibody–aptamer arrangement in this work offers good disease discrimination.

Overall, our work has identified a new PD biomarker that includes measuring aggregate morphology. It also provides a generic workflow for single-molecule biomarker discovery based on measuring both the concentration and the morphology of aggregates. In comparison to the established methods, this assay not only requires less sample volume (10 μL per patient) and consumables (∼15 ng detection antibody per patient) but is able to characterize the sample at much higher details. The assay is currently performed on a small glass coverslip (26 mm × 76 mm) due to the PEGlytion surface chemistry, so there is the opportunity to further improve the throughput. dSTORM imaging is also still time-consuming and requires further optimization for use in clinical practice. Novel real-time super-resolution techniques like structural light illumination^50^ have the potential to image aggregates more efficiently allowing larger-scale studies. The next-generation platform that we are currently developing incorporates enhanced surface chemistry alongside a robotic handling system. This advance should tackle the limitations observed in this study and has the potential to create a user-friendly platform that demands less expertise.

## Supporting Information Available

Extended details of method and materials, extended details of data analysis, extended statistics of the data, and assay validation (PDF)

## Notes

Figure 1 was produced by BioRender.com (license NO. BX25WG80V0).
