# Correction to “Imaging Protein Aggregates in Parkinson’s Disease Serum Using Aptamer-Assisted Single-Molecule Pull-Down”

**DOI:** 10.1021/acs.analchem.3c05050

**Published:** 2023-11-17

**Authors:** Yu P. Zhang, Evgeniia Lobanova, Derya Emin, Sergey V. Lobanov, Antonina Kouli, Caroline H. Williams-Gray, David Klenerman

In the original version of this article, an error was identified in Figure 5D–G. The morphology quantification of α-syn aggregates in Parkinson’s disease serum should have included 20 PD and 20 control samples, instead of 10 PD and 10 control samples. This figure was correct in the initial version of the manuscript but mistakenly replaced during the phase of preparing high-resolution figures. The results and conclusions of this paper were not affected by this correction.

**Figure 5:**
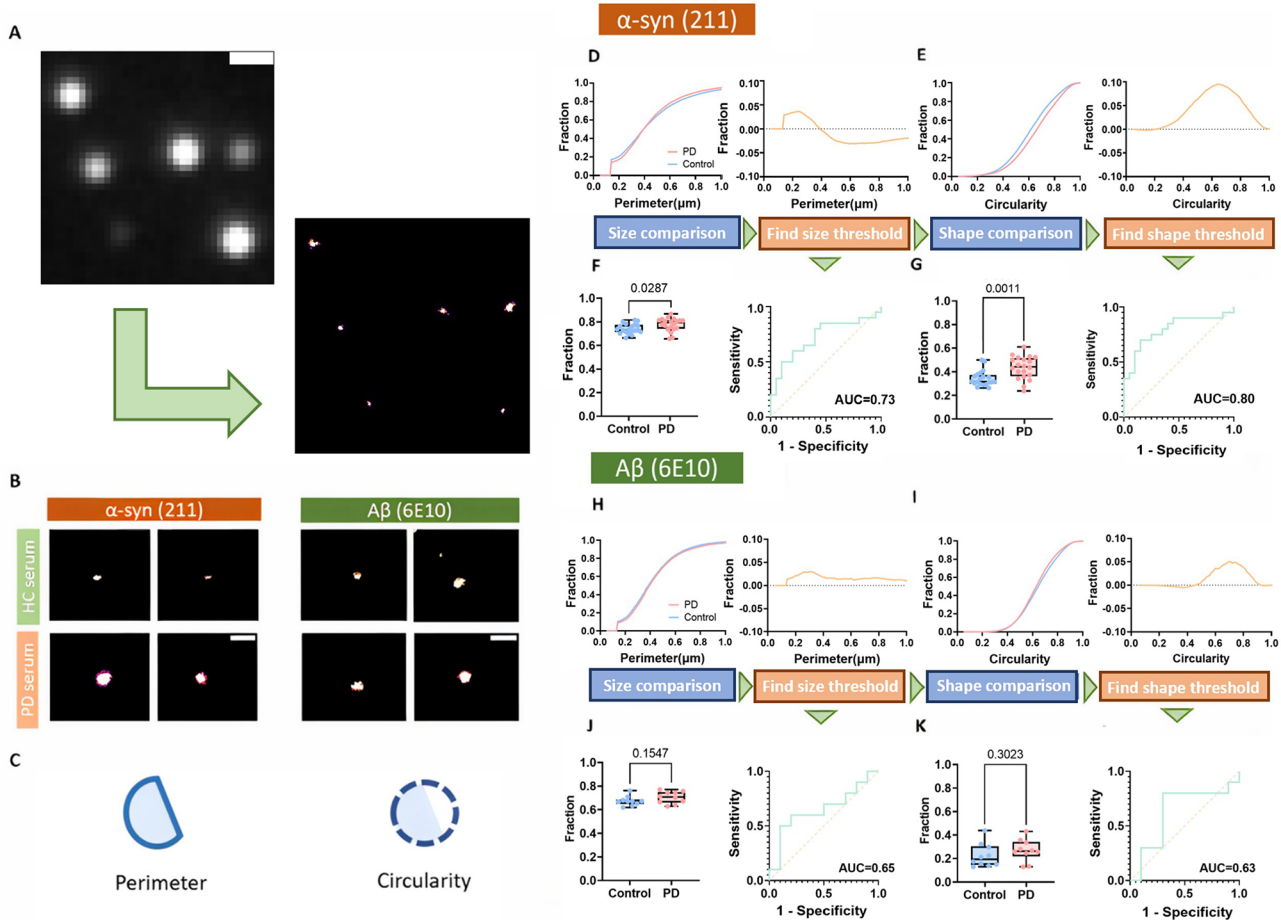
Morphology analysis of α-syn and Aβ aggregates in PD compared to control serum using dSTORM. (A) Diffraction-limited image of the serum sample and corresponding dSTORM image. Using dSTORM, the finer morphologic information masked by the diffraction limit can be revealed. For this representative image, AF-647-211 antibody was used to visualize α-syn aggregates in serum. The scale bar is 0.75 μm. (B) Examples of super-resolved aggregates in serum samples. The scale bar is 0.5 μm. (C) Two parameters (perimeter and circularity) were used to quantify morphological information. (D–G) The cumulative perimeter/size distribution of α-syn for two groups (*n* = 20 for PD, *n* = 20 for controls). α-syn aggregates in PD serum are larger and rounder than those in control serum. The cumulative difference (control-PD) determines the optimal morphology thresholds. The discrimination performance is presented by ROC analysis and *t* tests, showing its potential as a discriminator. (H–K) An identical workflow from (D–G) was applied to Aβ (*n* = 10 for PD, *n* = 10 for controls). A smaller morphological difference between PD and the control was observed for Aβ. Further ROC and *t* tests suggest that these parameters are not sufficient to serve as a discriminator.

