# Supplementary material for: Imaging Protein Aggregates in Parkinson’s Disease Serum Using Aptamer-Assisted Single-Molecule Pull-Down
Source: Anal Chem. 2023 Oct 2;95(41):15254–63. doi: 10.1021/acs.analchem.3c02515 (PMC10585954; doi:10.1021/acs.analchem.3c02515)
Supplement: Supplementary file 1 — ac3c02515_si_001.pdf [file ac3c02515_si_001.pdf]

Supporting information

Imaging protein aggregates in Parkinson's disease serum using aptamer-assisted single-molecule pull-down

Yu P. Zhang[1,2]*, Evgeniia Lobanova[1,2]*, Derya Emin[1,2]*, Sergey V. Lobanov[3], Antonina Kouli[4], Caroline H. Williams-Gray[4], and David Klenerman[1,2†]

***These authors contributed equally to this work**

†Correspondence author. Email: dk10012@cam.ac.uk

Affiliations

1 Department of Chemistry, University of Cambridge, Lensfield Road, Cambridge CB2 1EW, United Kingdom

2 UK Dementia Research Institute at Cambridge, Cambridge CB2 0XY, United Kingdom

3 Medical Research Council Centre for Neuropsychiatric Genetics and Genomics, Cardiff University, Cardiff CF24 4HQ, United Kingdom

4 Department of Clinical Neurosciences, University of Cambridge, Cambridge CB2 0PY, United Kingdom

Contents

1. Extended details of method and materials

2. Extended details of data analysis

3. Extended statistics of the data

4. Assay validation

Extended details of method and materials

Antibodies and aptamer

Biotinylated and Alexa 647 bi-modified T-SO508 aptamer (GCCTGTGGTGTTGGGGCGGGTGCGT) was purchased from ATDBio (Southampton, UK) and purified by high-performance liquid chromatography (HPLC). Biotinylated control aptamer (GGGTTAGGGTTAGGGTTAGGGTTT), which also forms a similar G-quadruplex structure like T-SO50823,32, was purchased from the same company and used in control experiments (See SI). The aptamer recognises β-sheet structure and is specific to both α-syn and Aβ aggregates 23. Alexa 647 conjugated anti-α-syn anti-body (211) was purchased from Santa Cruz biotech (Cat. SC-12767 AF647) and recognises amino acid 121-125 of human α-synuclein. Alexa 647 and 488 conjugated anti-Aβ antibody (6e10) targeting Aβ amino acids 1-16 was purchased from Biolegend (Cat. No. 803020 and No. 803013). For the biotinylated antibodies used in experiments (see SI), the biotinylated 6e10 antibody was purchased from Biolegend (Cat. No. 803007), and the biotinylated 211 antibodies were generated using a commercial kit (Mix-n-Stain™, 92444). Unconjugated 211 antibodies were purchased from Santa Cruz biotech (Cat. SC-12767). Alexa 647 Mouse IgG1 (AB_2539542, Invitrogen), Bovine Serum Albumin (B9000S, New England biolabs) were also purchased commercially.

Synthetic α-syn and Aβ aggregates

α-syn monomers are expressed and purified from E.coli following a published protocol 44. $A\beta_{1\text{-}40}$ was purchased commercially (Bio Trend, AS-24235) and purified according to the supplier's protocol. The initial protein concentrations were measured using a BCA assay (Thermo fisher, 23225). To aggregate these two species, α-syn was diluted into 70μM using filtered PBS (pH 7.4, 0.02μm Whatman, 6780-1302 filters) and incubated at 37℃ under 200 rpm constant shaking for 3 days. The Aβ was diluted into 20μM using PBS using the same method and incubated under the same conditions for 2 days without shaking. To generate the sonicated aggregates, incubated aggregates (both α-syn and Aβ) were pulse-sonicated using a tip sonicator (QSonica Q125, tip size 1/8 inch). Each pulse includes a 5 second on-status and 15 second off-status and 3 cycles are conducted. The power was set to 40% during the sonication.

Serum samples preparation

Blood was collected from participants by venepuncture using 7.5 ml S-Monovette tubes, and samples were left to clot at room temperature for 15 minutes before centrifuging at 2000 rpm for 15 minutes at room temperature. The supernatant (serum) was collected and stored at -80 ℃ until use. In order to minimise the impact of repeat freeze-thawed cycles, following the first thaw of samples, single-use 11μL aliquots were prepared in protein low bind tubes (Protein LoBind® Tubes 0.5mL, Eppendorf).

Coverslip preparation

Coverslips were prepared following previously published works. 12,32Glass coverslips (26x76 mm, thickness #1.5, VWR, Cat. No. MENZBC026076AC40) were sequentially bath sonicated (Ultrasonic cleaner USC100T, VWR) with MQ water (18.2-MΩ cm−1), Acetone, and Methanol for 10 minutes each to remove contaminants. Cleaned coverslips were then etched by 1M KOH for 20 min under the same sonication condition before rinsing with MQ water and then Methanol. Nitrogen flow is used to remove the Methanol residues and the dried coverslips are then cleaned with argon plasma for 15 minutes (Femto Plasma Cleaner; Diener Electronic). The cleaned coverslips were then silanized with 5ml of 3-aminopropyl triethoxysilane (Fisher Scientific UK, cat. no. 10677502), 8.3ml of Acetic acid in 166 ml of methanol for 20 minutes. A pulse sonication of approximately 60 seconds was performed at the beginning and midpoint of the process. The salinized coverslips were then sequentially rinsed by Methanol, MQ water, Methanol and dried with nitrogen flow. 50-well PDMS chamber gasket (cut from a CultureWell chambered cover-glass, Sigma, cat. no. GBL103350-20EA) was attached to the processed coverslips. PEGylation of coverslip was performed in every well. Each well was treated by adding 9µl of a 100:1 aqueous mixture of methoxy- (110mg/ml, ~22mM, Mw ~5,000; Laysan Bio Inc., cat. no. MPEG-SVA-5000) and biotin-terminated (1.1mg/ml, ~220µM, Mw ~5,000, Laysan Bio Inc., cat. no. Biotin-PEG-SVA-5000) PEGs. 1µl of 1M NaHCO3 (pH 8.3) was added after the loading of PEG solution to speed up the reaction. The reaction was performed overnight at room temperature in a humid chamber. Coated coverslips were then rinsed with MQ water and dried with Nitrogen flow. A second-round PEGlytion was then performed to enhance the passivation. 9µl of another smaller methoxy-terminated PEG (10mg/ml, 30mM, MS(PEG)4 methyl-PEG-NHS-Ester; ThermoFisher, cat. no. 22341) was added to each well before further loading of 1µl of 1M NaHCO3 (pH 8.3). The reaction was performed overnight at room temperature in a humid chamber. Coated coverslips were then rinsed with MQ water and dried with Nitrogen flow. Co-verslips were stored in a desiccator at −20 °C until needed.

Extended details of data analysis

Analysis of diffraction-limited imaging

limited single-molecule fluorescence images with only two intuitive parameters, which are automatically determined in the software using statistical approaches. The analysis includes four main steps: (i) global background subtraction; (ii) convolution with the point spread function; (iii) determination of a global intensity threshold; (iv) finding aggregates (see the description of algorithms below). As a validity control, we demonstrated that our analysis method is linear in concentration using dilution series of sonicated in-vitro α-syn and compared it with the Find Maxima ImageJ tool on the same dataset. In each case, we calculated the divergence from a linear curve using a sum of squared errors (SEM) and our method gave 4 times smaller error (Figure S1(A)) than the Find Maxima ImageJ (Figure S1(B)). We also demonstrate that our cluster analysis is capable to recognise both small and long complex-shaped aggregates from the SiMPull images of sonicated in-vitro α-syn (Figure S1(C)) and late-stage $A\beta_{40}$ fibrils (Figure S1(E)) compared to a multiple counting of long aggregates with the Find Maxima ImageJ (see Figure S1(F)).

(I) global background subtraction: The global background B_{ij} is initially calculated by fitting the intensities of the diffraction limited images I_{ijk} with a normal distribution $\mathcal{N}(B_{ij},\sigma_{ij}^2)$ pixel by pixel. Here, B_{ij} and σ_{ij}^2 are the unknown mean and variance to be estimated, the subscripts i and j label the pixel, and the index k enumerates the diffraction limited images. The parameters of a normal distribution (mean and variance) are estimated by equating the intensities of the observed (experimental) and normal theoretical distributions at the 25th and 75th percentiles. Since the intensity histogram consists of a prevalent background peak and a long tail represented by the aggregates (the outliers of the normal background distribution), we aim to separate them from each other. For this, we iteratively filter out intensities with right-sided p-value below $0.05/N_s$, where N_s is the number of diffraction limited images (see Figure S2).

We then smooth the global background B_{ij} by approximating it as a sum of 2D smooth function and normally distributed noise $\mathcal{N}(0,\sigma^2)$. Pixels with high noise level (two-sided p-value is below the Bonferroni-corrected threshold of 0.05) are iteratively removed. We use the first 5 harmonics of the Fourier sine and cosine series augmented by a linear function to model the smooth background.

(II) convolution with point spread function: after the global background subtraction $\Delta I_{ijk} = I_{ijk} - B_{ij}$, we convolute the background-free intensity with an Airy disk (the central region of the 2D point spread function) $I_{ij}^{\mathrm{Airy}} = \left(2J_1(r_{ij})/r_{ij}\right)^2$ for $r_{ij} < \pi$ and $I_{ij}^{\mathrm{Airy}} = 0$ otherwise. Here, $J_1(x)$ is the Bessel function of the first order, $r_{ij} = 2\pi/\lambda \cdot \mathrm{NA} \cdot d\sqrt{i^2 + j^2}$, λ is the wavelength of light, NA is the numerical aperture, and d is the pixel size. This step allows to reduce the noise level since information in neighbouring pixels is now used.

(III) determination of a global intensity threshold: to find the global intensity threshold, we use a similar approach as described above (Figure S3(A)). We assume the elements of the intensity matrix ΔI_{ijk} are normally distributed and iteratively remove elements with right/left-sided p-value below $0.05/N$. Here, N is the number of elements of the intensity matrix ΔI_{ijk}, i.e. $N=N_p N_s$ where N_p is the number of pixels. The intensity, at which the right-sided p-value is $0.05/N$, is selected as a global intensity threshold. It is worth noting that we optionally multiply the global intensity threshold by 2 to improve the detection accuracy.

(IV) finding aggregates: finally, we select pixels with intensity above the global intensity threshold and group them into clusters (aggregates) (Figure S3 (B,C)). Two clusters with peak intensities I_1 and I_2 are considered different if they cannot be connected with a path, intensity along which is always higher than $F \cdot \min(I_1, I_2)$. Here, F is the resolution factor (we used $F = 0.5$ as a default value).

In this study, we cut the first 10 frames to avoid blurry images due to the focusing stabilisation. Then we set the threshold of 2 for all serum samples. For in-vitro samples, which mainly present in the supplementary, we used threshold of 2 for α-syn and 1 for Aβ. We used peak sensitivity of 0.3 for all samples. When calculating the intensity, the sum of intensity in the processed data was used as a metric.

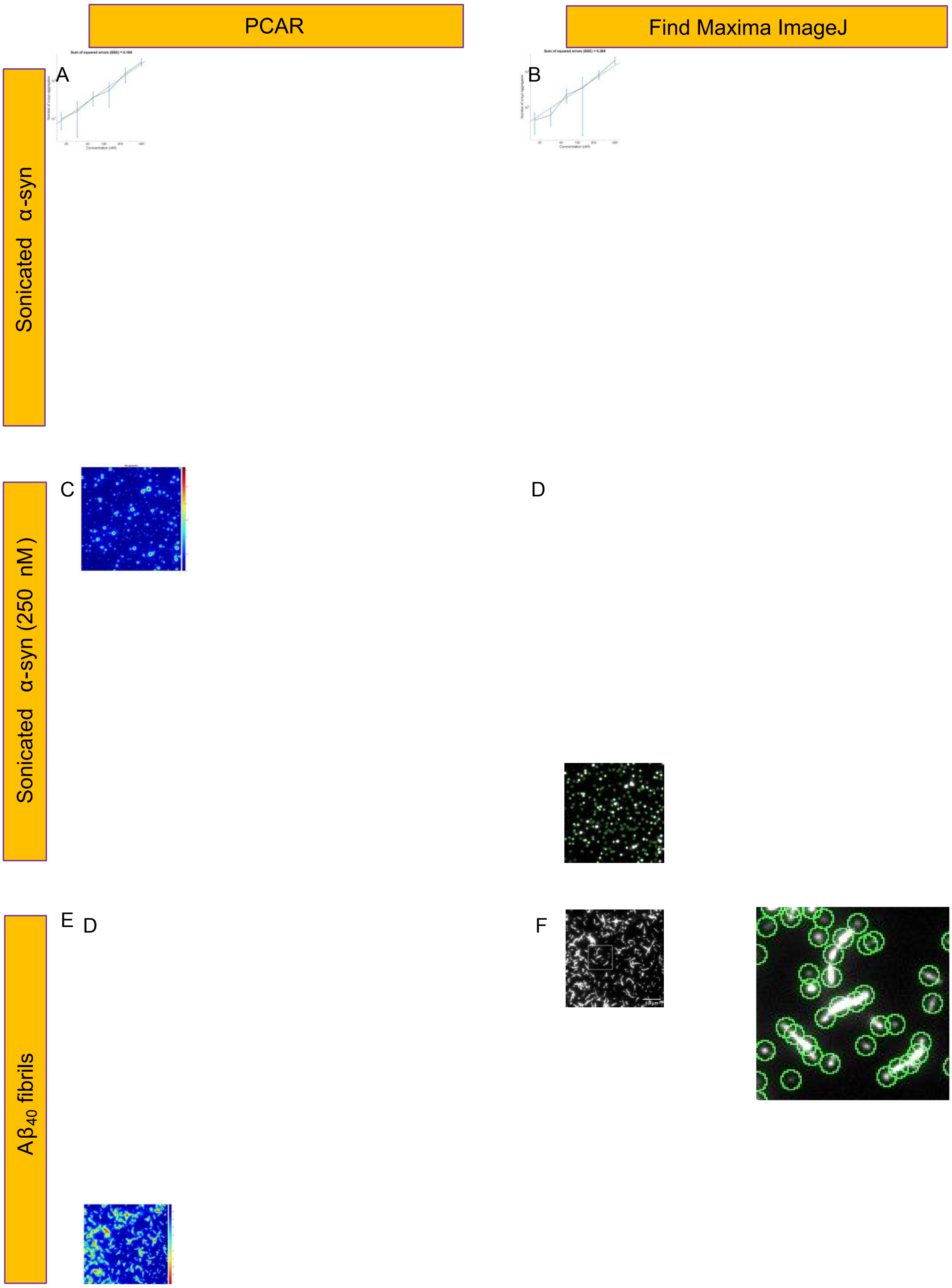

Figure S1. Validation of PCAR and its comparison with the Find Maxima ImageJ tool. Linearity in concentration of PCAR (A) vs the Find Maxima ImageJ (B) was confirmed using a dilution series of sonicated in-vitro α-syn aggregates . The superior performance of cluster analysis using PCAR (C, E) over the Find Maxima ImageJ tool (D, F) on the SiMPull images of sonicated in-vitro α-syn aggregates (C, D) and late-stage Aβ_{40} fibrils (E, F) is shown. This demonstrates the capability of PCAR to recognise both small and long complex-shaped aggregates in contrast to the multiple counting of long aggregates with the Find Maxima ImageJ.

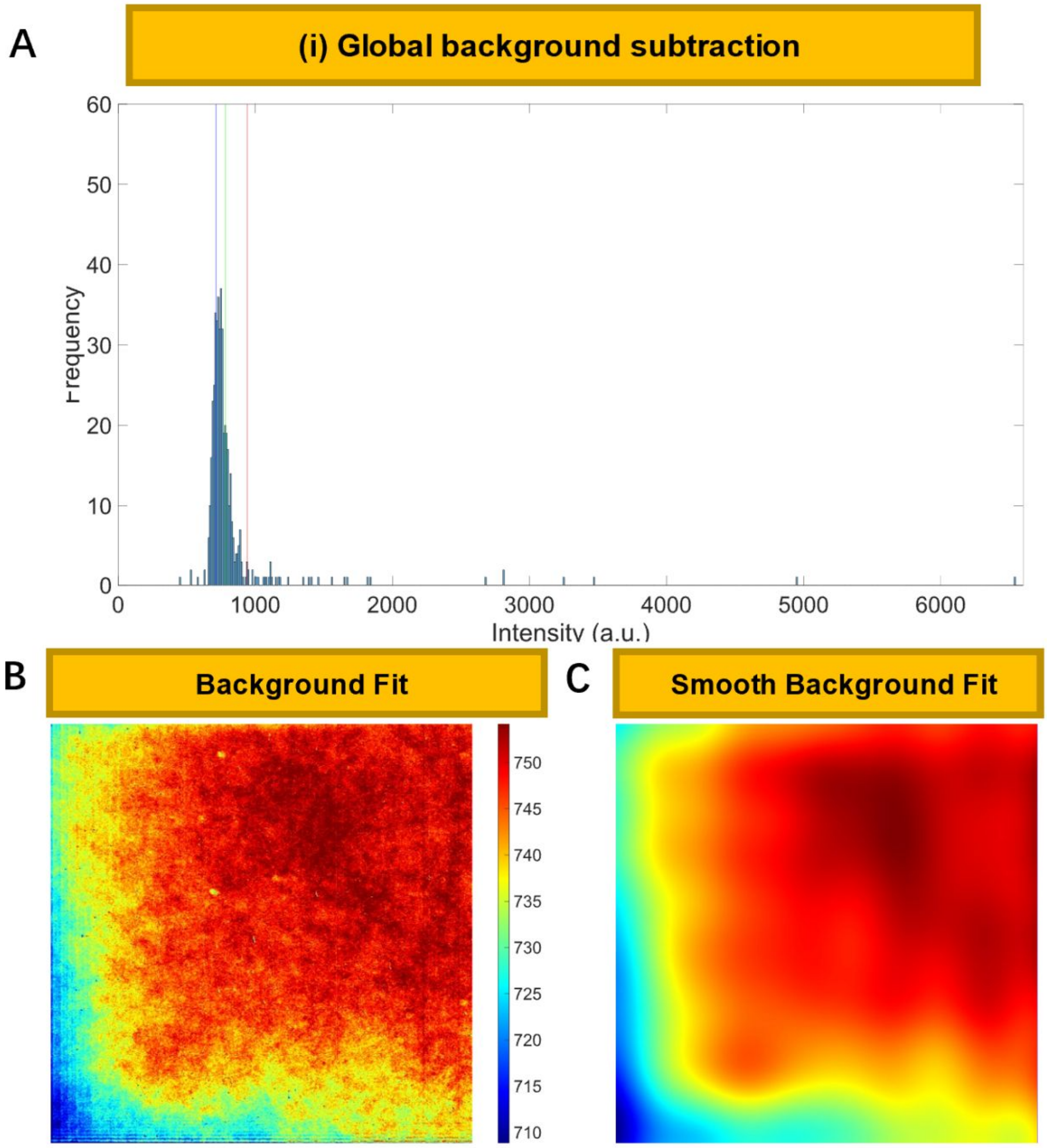

Figure S2. Global background subtraction with the PCAR (GBS-PCAR). (A) Demonstration of the background subtraction procedure using SiMPull data with N_s = 509 images. The intensity histogram reconstructed from the experimental data for individual pixels is used to determine the intensities of the 25th (blue line) and 75th (green line) percentiles of the observed distribution. The parameters of normal theoretical distribution attributed to background are calculated based on these values and then used to iteratively find the threshold (red line) with the right-sided *p*-value of $0.05/N_s$. The defined threshold is used to filter out the outliers of the observed distribution represented by the actual single-molecule spots so that the low-intensity background is accurately estimated. (B,C) Exact background fit (B) and final background fit after smoothing (C) found from the GBS-PCAR.

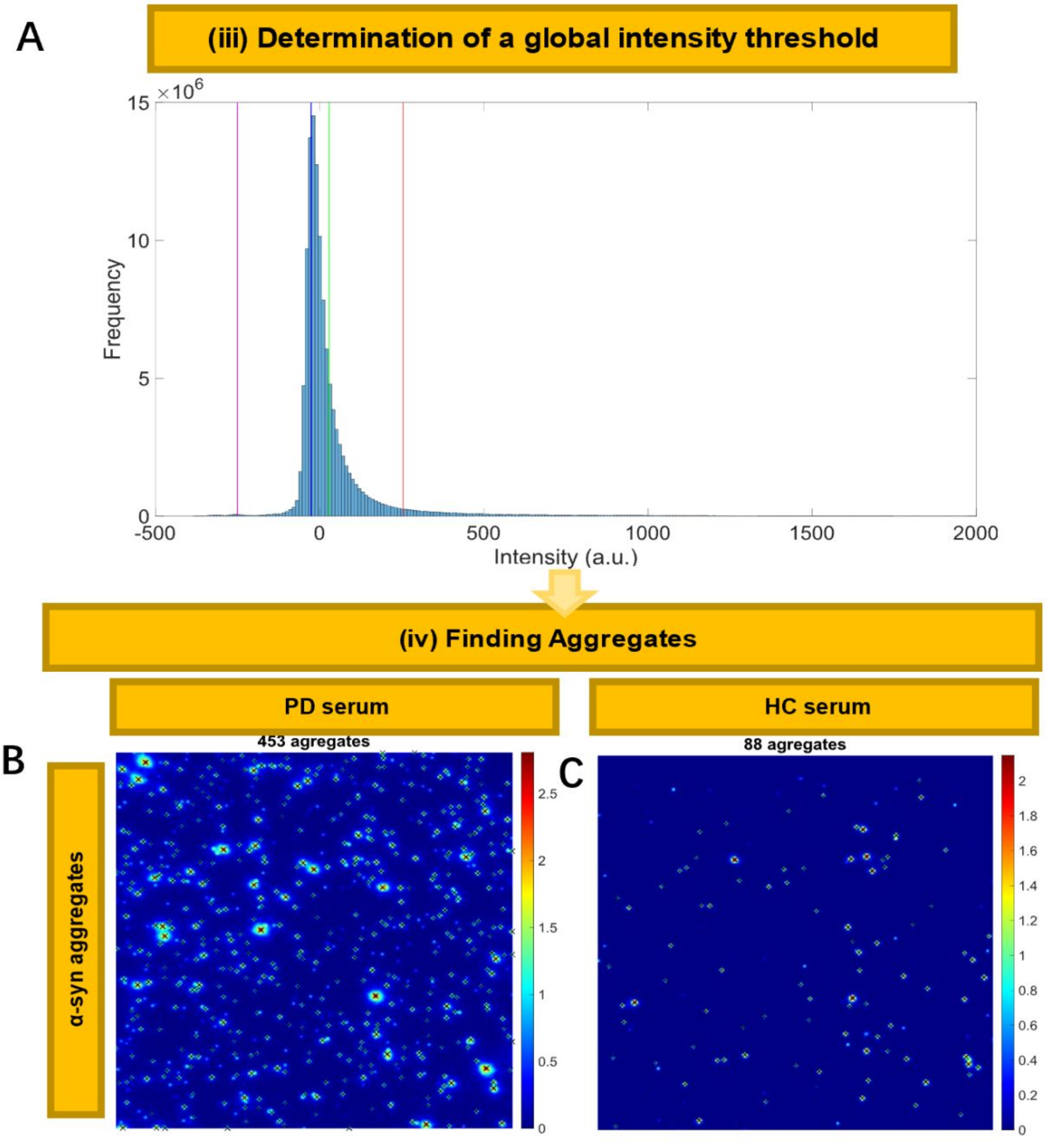

Figure S3. Applying the global intensity threshold and cluster analysis to the experimental single-molecule data in the PCAR software. (A) The total intensity histogram of the background-free data retrieved after the global background subtraction in Fig. 2 is used again to determine the intensities of the 25th (blue line) and 75th (green line) percentiles. We assume that the non-specific binding spots have normal distribution, the parameters of which could be calculated from the values above and then used to iteratively find the global intensity threshold (red and magenta line) as the intensities with the right- and left-sided p-value of 0.05/(total number of pixels). The defined threshold is used to filter out the outliers of the observed distribution represented by actual aggregates so that non-specific spots are separated. (B,C) Representative SiMPull images of detected α-syn aggregates (crosses) in PD (B) and HC (C) serum found from the PCAR analysis. Colour encodes intensity I in the units of the threshold I_{thr}. The scale is linear from 0 to 1 (I/I_{thr}) and logarithmic ($1 + \log_{10}(I/I_{\text{thr}})$) above 1.

Super resolution imaging analysis

As stated in the methods section, the analysis tool used in this study is based on relevant published works. Briefly speaking, to avoid blurring images collected during focusing stabilisation, first 200 frames was cut before further analysing. The localisations are then identified using ThunderSTORM[1]. The drift correction was calculated using a feeding mean shift algorithm[2] with localisations. Once corrected, the localisations were filtered using a localisation merge and density filter and then used to reconstruct the super-resolution images. Morphology analysis[3] was performed based on these images. The key parameters used in this work can be found in our code.

Extended statistics of the data

Table S1. Details of K-S test on the datasets in which statistical tests were performed

Sample pair	KS distance		P value (KS)		Normality	
	Control	PD	Control	PD	Control	PD
α-syn level	0.1409	0.1968	>0.1000	0.0410	Yes	No
Aβ level	0.2344	0.2391	0.0053	0.0040	No	No
α-syn/(α-syn+ Aβ) ratio	0.1422	0.1409	>0.1000	>0.1000	Yes	Yes
α-syn+ Aβ level	0.1974	0.1879	0.0398	0.0623	No	Yes
α-syn intensity	0.2331	0.2163	0.0057	0.0150	No	No
Aβ intensity	0.1992	0.2576	0.0364	0.0012	No	No
Distinctive α-syn ratio (Peri.)	0.1530	0.1373	>0.1000	>0.1000	Yes	Yes
Distinctive α-syn ratio (Peri.+Circ.)	0.2013	0.1123	0.0328	>0.1000	No	Yes
Distinctive Aβ ratio (Peri.)	0.2375	0.1816	>0.1000	>0.1000	Yes	Yes
Distinctive Aβ ratio (Peri.+Circ.)	0.1975	0.1984	>0.1000	>0.1000	Yes	Yes
Combined discriminator (C1)	0.3773	0.2257	0.0006	>0.1000	No	Yes
α-syn/(α-syn+ Aβ) ratio (Validation)	0.2332	0.2222	>0.1000	>0.1000	Yes	Yes
α-syn intensity (Validation)	0.1984	0.2371	>0.1000	>0.1000	Yes	Yes
Aβ intensity (Validation)	0.1645	0.1948	>0.1000	>0.1000	Yes	Yes
Combined discriminator 2 (Validation)	0.2800	0.2186	0.0404	>0.1000	No	Yes

Assay validation

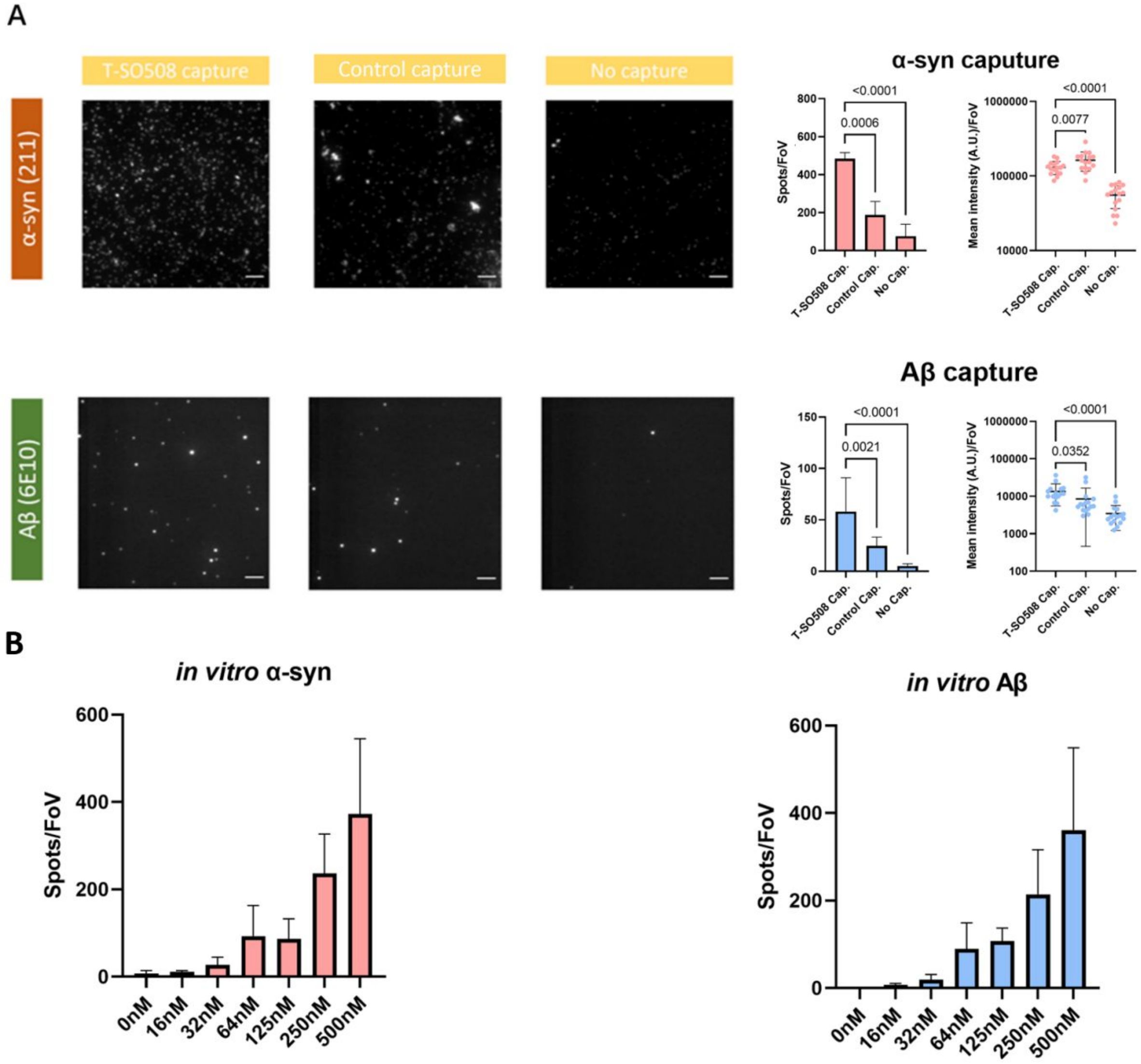

Figure S4. Assay validation. **A**. In-vitro α-syn (500nM) and Aβ (125nM) aggregates were applied with correct capture (T-SO508), control capture (G-quadruplex) and non capture aptamer on surface. Correct setup produced highest single molecule counting, reflecting functional aptamer capture. The intensity of captured single molecules also showed that the aptamer capture has a size preference over non-specific sample binding. S.D. n=16 from 16 fields of views. Ordinary one-way ANOVA (for data with normal distribution) and Kruskal-Wallis methods (for data with non-normal distribution) were used for statistical tests. **B.** Calibration curve using synthetic α-syn and Aβ aggregates. Aggregates concentration here represents monomer equivalent. The contrast of the images has been adjusted to the same level for the same protein aggregates. S.D. n=20 from 20 fields of views. Scale bar: 5µm.

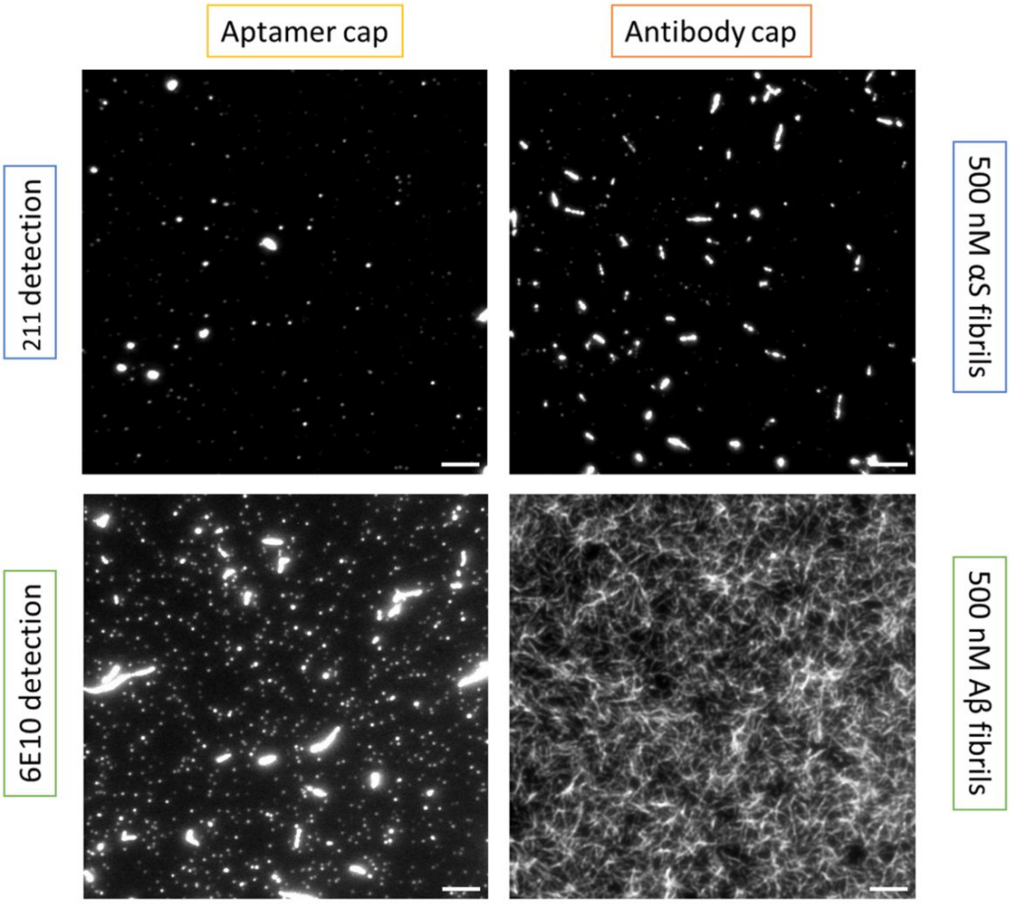

Figure S5. Comparison of binding specificity between the antibody surface and aptamer surface. The aptamer surface only shows a strong affinity to smaller species rather than mature fibrils. The contrast of the images has been adjusted to the same level for the same protein aggregates. Scale bar: 5 µm.

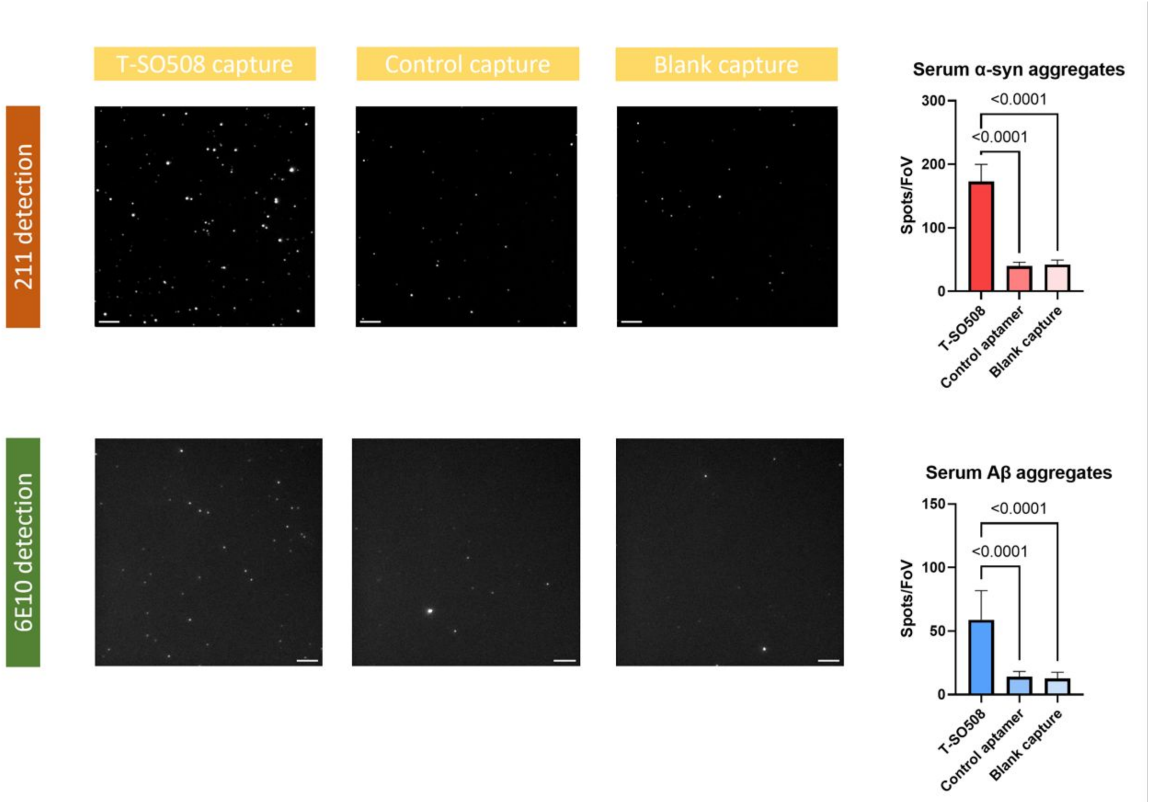

Figure S6. Assay capture specificity control with human serum samples. The assay only shows a strong affinity to aggregates in serum with correct capture aptamer. S.D. n=16 from 16 fields of views. Scale bar: 5 μm.

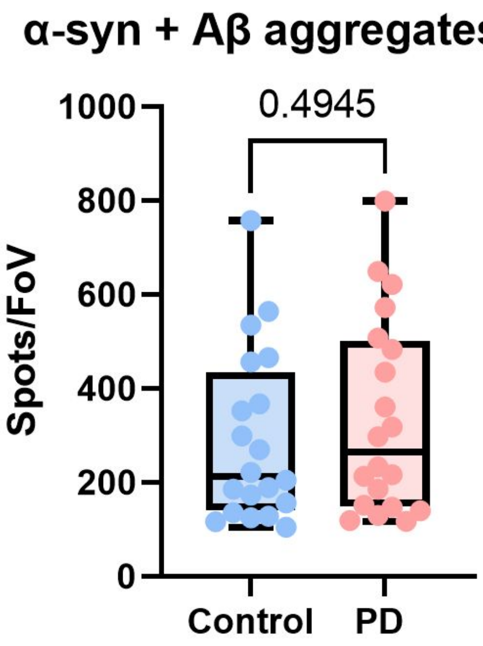

Fig. S7. Data of number of total aggregates (α-syn and Aβ) detected in individual samples. (n=20 for both control and PD)

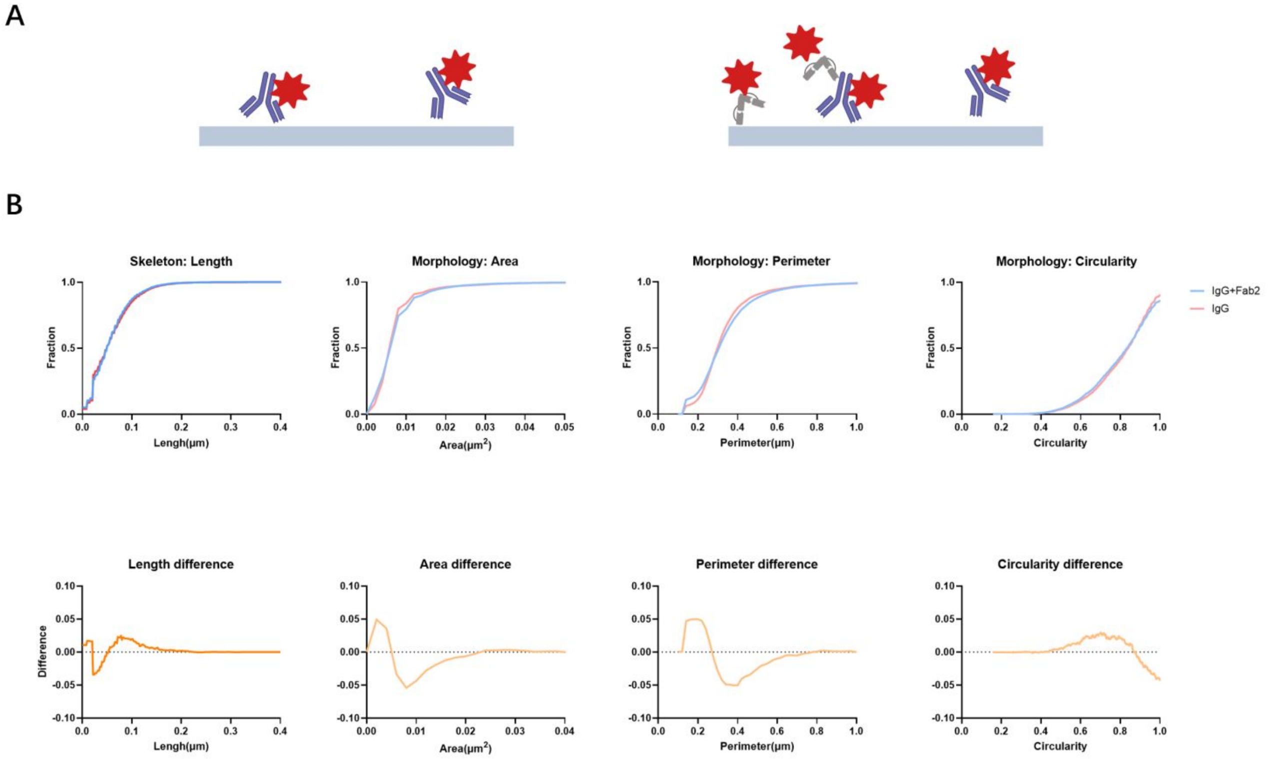

Figure S8. Verification of morphological analysis via dSTORM imaging. **A**. dSTORM imaging was performed in two conditions: one with only Alexa 647 conjugated IgG antibodies and one with an additional secondary Alexa 647 conjugated Fab_2. The size difference between the IgG and IgG+ Fab_2 is around 20nm **B.** We analysed the morphological information of these images showing that the difference could be identified via morphology analysis. The IgG + Fab_2 mixture has a larger population of big and small objects, which corresponds to smaller Fab_2 fragments and big IgG + Fab_2 complex. The area and perimeter characterise the size of objects while circularity stands for shape. Since area and perimeter offers equivalent information, we used the combination of perimeter (for better resolution) and circularity to characterise the aggregates. The analysis done by skeletonization (as shown in the left end of the panel), is less efficient in obtaining the difference between 2 samples.

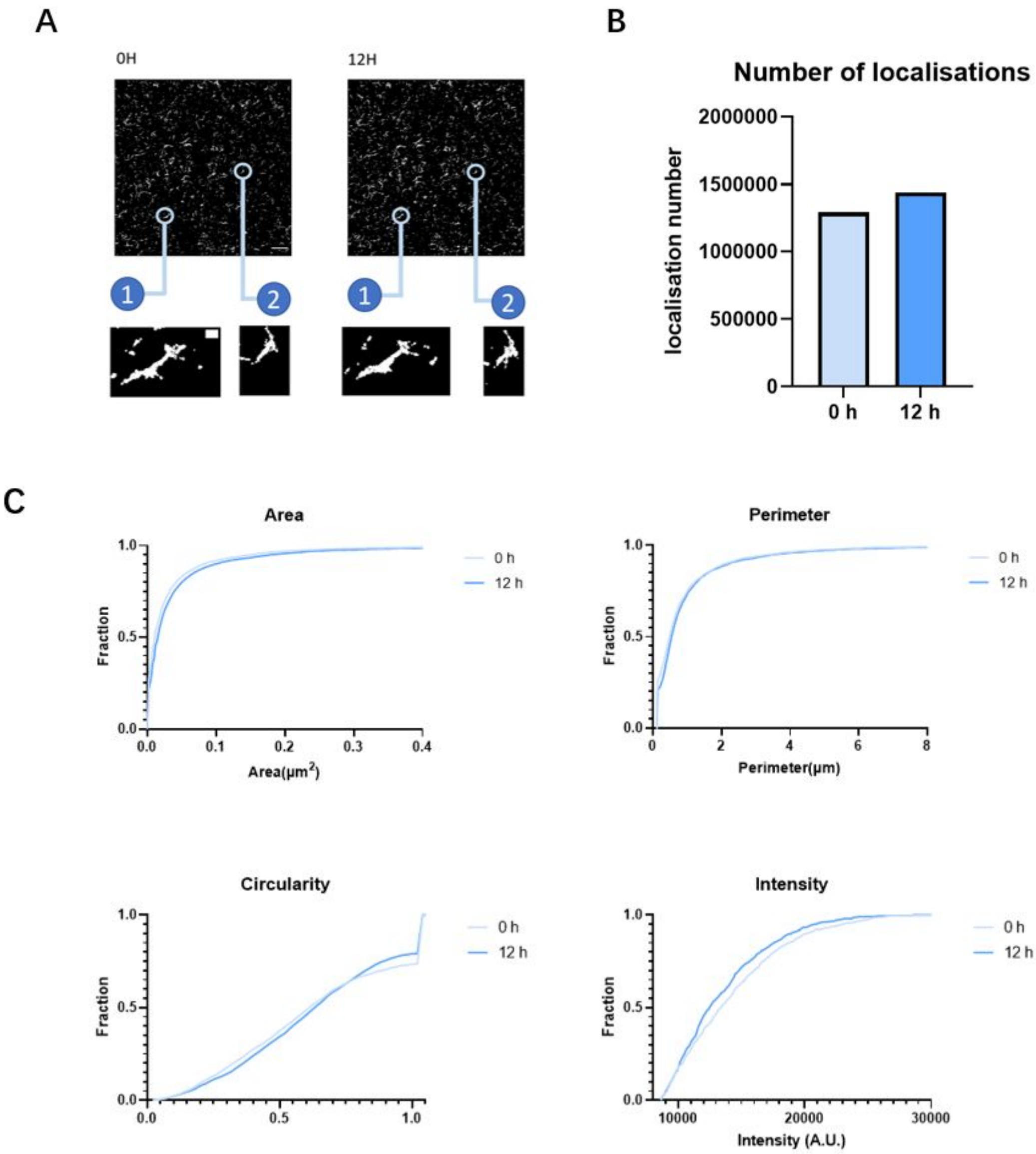

Figure S9. Stability of dSTORM imaging. **A**. Two identical field of views collected at 0h and 12h. The imaging remains the same 12h after buffer loading. Scale bar: 5 µm (upper panel), 0.5 µm (lower panel). **B**. Localizations identified at 0h and 12h. **C**. Statistics of morphological information collected from super resolution image in panel A. Unlike the brightness/intensity statistics, the distribution of morphological features are very stable. Only circularity information showed a larger variance due to the complicated morphology of fibrils. We collected 9000 frames for this experiment and cut first 3000 before further processing due to the strong signal.

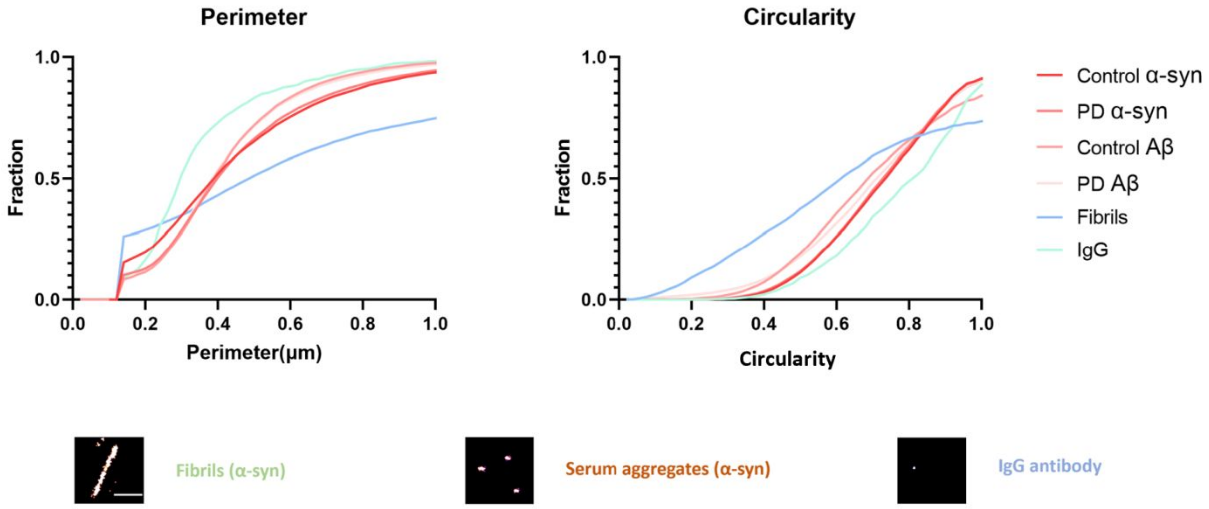

Figure S10. Cumulative distributions of morphological parameters of aggregates and antibodies. The signal collected from antibodies (in blue) is smaller and rounder while the signal collected from fibrils is larger and elongated (in green). The cumulative distribution of aggregates detected in serum lie between the two. In order to image the fibrils on the surface, 211 capture antibody was used instead of T-S508 aptamer. Scale bar: 0.5 μm.

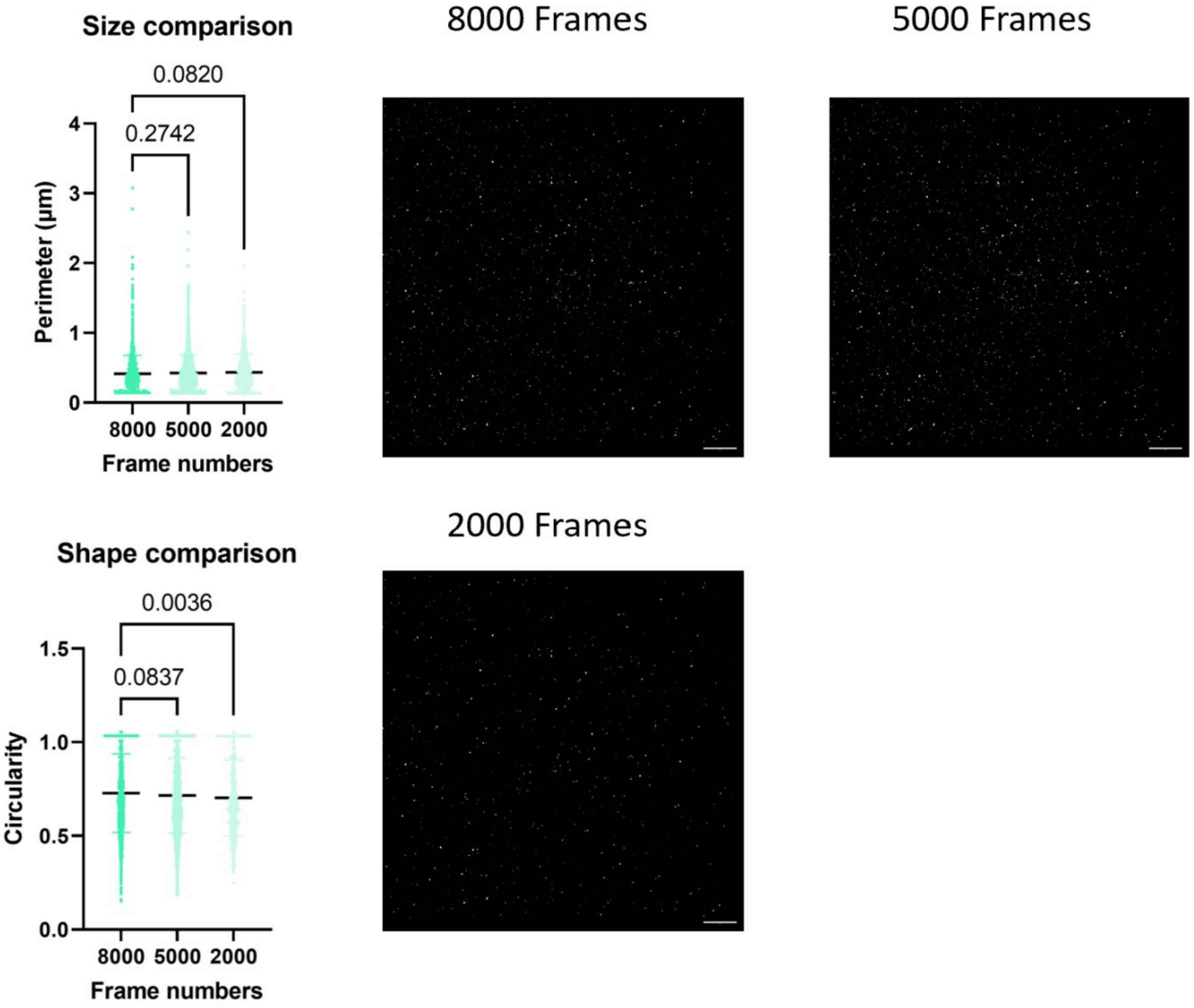

Figure S11. Comparison between dSTORM images reconstructed from different number of frames. Stable morphological information of aggregates is observed from reconstructed images with a frame number higher than 5000. Ordinary one-way ANOVA was used in the statistic test. Scale bar: 5 μm.

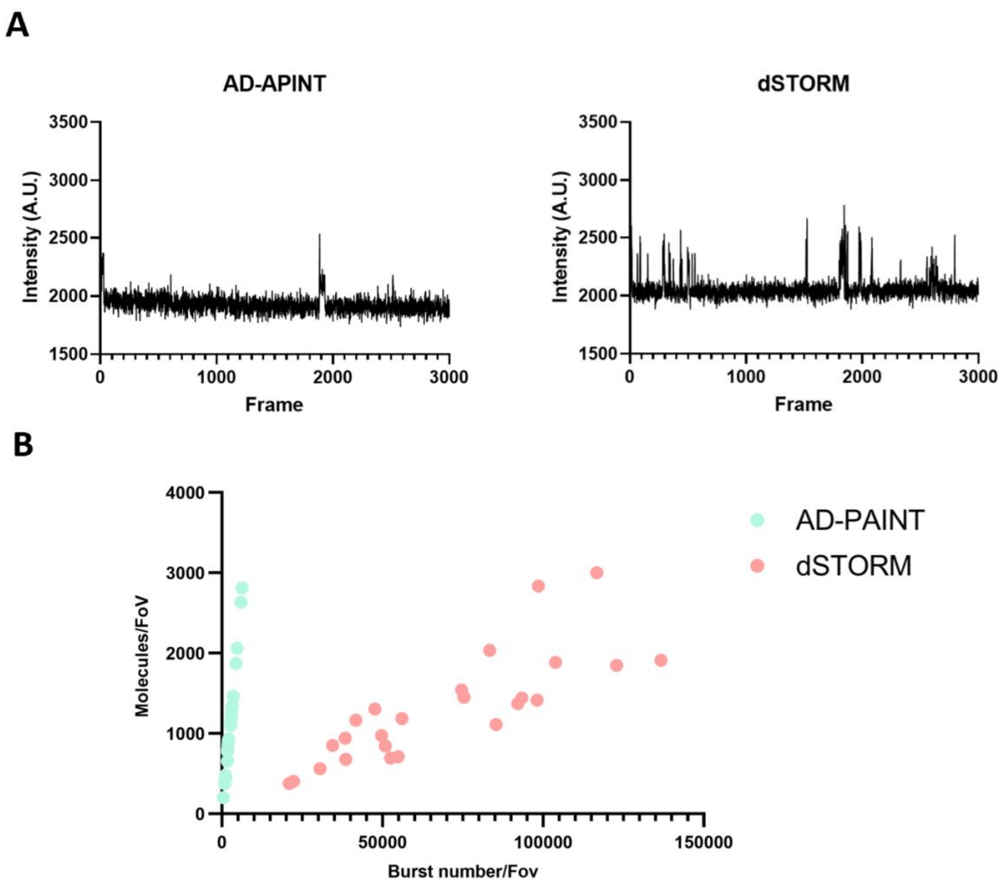

Figure S12. Comparison between single molecule burst collected with dSTORM and AD-PAINT. A. Representative traces of a single-molecule in both AD-PAINT and dSTORM experiment. The super-resolution images of single molecules are constructed with these bursts. Molecules require sufficient number of bursts for accurate reconstruction. AD-PAINT data is from our previous work[4] and dSTORM data is from this work. **B.** Number of reconstructed molecules and number of bursts used to constructed in AD-PAINT and dSTORM. Each data point represent one image(FoV) reconstructed with two methods. 20 random images were selected for comparison.

Supplementary references

(1) Ovesný, M.; Křížek, P.; Borkovec, J.; Švindrych, Z.; Hagen, G. M. ThunderSTORM: A Comprehensive ImageJ Plug-in for PALM and STORM Data Analysis and Super-Resolution Imaging. *Bioinformatics* 2014, *30* (16), 2389–2390. https://doi.org/10.1093/bioinformatics/btu202.

(2) Fazekas, F. J.; Shaw, T. R.; Kim, S.; Bogucki, R. A.; Veatch, S. L. A Mean Shift Algorithm for Drift Correction in Localization Microscopy. *Biophysical Reports* 2021, *1* (1), 100008. https://doi.org/https://doi.org/10.1016/j.bpr.2021.100008.

(3) Legland, D.; Arganda-Carreras, I.; Andrey, P. MorphoLibJ: Integrated Library and Plugins for Mathematical Morphology with ImageJ. *Bioinformatics* 2016, *32* (22), 3532–3534. https://doi.org/10.1093/bioinformatics/btw413.

(4) Lobanova, E.; Whiten, D.; Ruggeri, F. S.; Taylor, C.; Kouli, A.; Xia, Z.; Emin, D.; Zhang, Y. P.; Lam, J. Y. L.; Williams-Gray, C. H.; Klenerman, D. Imaging Protein Aggregates in the Serum and Cerebrospinal Fluid in Parkinson's Disease. *Brain* 2021. https://doi.org/10.1093/brain/awab306.
